# Accelerating Sono‐Piezoelectric Charge Transport for Antibacterial Therapy and Bone Regeneration by Metal‐Deficient TiO_2_ Spin‐Polarization Effect

**DOI:** 10.1002/advs.202503186

**Published:** 2025-05-28

**Authors:** Chaofeng Wang, Shuilin Wu, Congyang Mao, Liguo Jin, Hanpeng Liu, Yufeng Zheng, Chunyong Liang, Shengli Zhu, Zhaoyang Li, Hui Jiang, Xiangmei Liu

**Affiliations:** ^1^ School of Health Science and Biomedical Engineering Hebei University of Technology Tianjin 300131 China; ^2^ School of Materials Science & Engineering Peking University Yiheyuan Road 5# Beijing 100871 China; ^3^ Biomedical Materials Engineering Research Center Hubei Key Laboratory of Polymer Materials Ministry‐of‐Education Key Laboratory for the Green Preparation and Application of Functional Materials School of Materials Science & Engineering State Key Laboratory of Biocatalysis and Enzyme Engineering Hubei University Wuhan 430062 China; ^4^ School of Materials Science & Engineering The Key Laboratory of Advanced Ceramics and Machining Technology by the Ministry of Education of China Tianjin University Yaguan Road 135# Tianjin 300072 China

**Keywords:** antibacterial, deep‐seated infections, mitochondrial fusion, piezoelectric charges, spin‐polarized TiO_2_

## Abstract

Sonodynamic therapy (SDT) is recognized as an effective method for treating deep tissue bacterial infections, but enhancing charge transfer to achieve efficient SDT remains a formidable challenge. Here, deficient TiO_2_ (DTO) is fabricated and Barium titanate (BTO) heterojunction with oxygen vacancies (OVs) and abundant Ti^3+^ species on a Ti substrate. The OVs in DTO not only narrow the band gap of TiO_2_ but also expedite the transfer of surrounding electrons to Ti^3+^. Meanwhile, the spin‐polarization effect induced by the unpaired spin electrons in Ti atoms can expedite the transfer of ultrasonic piezoelectric charges in BTO, enhancing surface sonocatalytic efficiency. Consequently, the DTO/BTO with US irradiation can eradicate 99.82% of *Staphylococcus aureus* and inhibit biofilm formation. Additionally, the microcurrent generated by ultrasound‐excited DTO/BTO enhanced mitochondrial fusion and promoted osteoblastic differentiation. The successful application of this ultrasound‐mediated DTO/BTO in treating bone infection defects offers an effective antibiotic‐free therapeutic strategy for deep‐seated infections.

## Graphical abstract

The deficient TiO_2_ (DTO) and Barium titanate (BTO) heterojunction treats osteomyelitis through SDT. The spin‐polarization effect induced by the unpaired spin electrons in Ti atoms can expedite the transfer of ultrasonic piezoelectric charges in BTO, enhancing surface sonocatalytic efficiency. Additionally, the microcurrent generated by ultrasound‐excited DTO/BTO enhanced mitochondrial fusion and promoted osteoblastic differentiation.

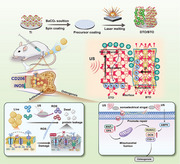

## 1 Introduction

Osteomyelitis is usually an inflammatory disease caused by Staphylococcus aureus (S. aureus) infection.^[^ ^1^, ^2^ ^]^ If left untreated, the bacteria will invade the surrounding bone tissues, triggering an inflammatory response that prevents bone regeneration and, in severe cases, can lead to necrosis of the bone tissue.^[^ ^3^, ^4^ ^]^ Currently, surgical debridement combined with high‐dose antibiotics is the main treatment strategy for clinical antibacterial.^[^ ^5^ ^]^ However, the misuse of antibiotics often leads to the emergence of bacterial resistance, which increases the difficulty of treatment; moreover, secondary infections during surgery may further affect bone tissue regeneration.^[^ ^6^, ^7^ ^]^ Therefore, the development of a noninvasive, highly effective antimicrobial strategy for deep tissue infections is essential. In recent years, some exogenous means, such as photothermal therapy (PTT), photodynamic therapy (PDT), and acoustic power therapy, have been widely used in antimicrobial therapy.^[^ ^8^ ^]^ For example, Zhang et al. used photodynamic removal of bacterial infections by synthesizing synthetically defective copper sulphide (CuS) under 808 nm excitation^[^ ^9^ ^]^; Shuai et al. synthesized a polydopamine nanorobot for synergistic antimicrobial use in PDT/PTT^[^ ^10^ ^]^; And Deng et al. designed a bioheterojunction that clears bacteria under NIR irradiation by generating ROS and thermally destroying the bacterial structure.^[^ ^11^ ^]^ However, due to the limited penetration ability of light, its application is also mainly focused on the therapeutic field of skin infections, which cannot cope well with deep tissue infections.

Sonodynamic therapy (SDT) has attracted much attention due to its deep tissue penetration, noninvasiveness, and flexibility and control.^[^ ^12^ ^]^ SDT mainly utilizes ultrasound (US) to produce a mechanical effect as well as a cavitation effect, which excites the separation of electron and hole pairs in the ultrasound sensitizers, which then induces a redox reaction between oxygen and water to produce reactive oxygen species (ROS) for sterilization.^[^ ^13^ ^]^ However, although titanium‐based implants possess excellent mechanical properties and biocompatibility, they still face significant challenges in terms of responsiveness under ultrasonic stimulation. First, there is a substantial acoustic impedance mismatch between titanium and the surrounding soft tissues, which limits the efficient transmission and utilization of ultrasound energy.^[^ ^14^ ^]^ Second, titanium alloys lack effective acoustic‐to‐electrical conversion capabilities necessary for activating signaling pathways related to bone regeneration. Regarding bacterial infections, conventional titanium alloys do not possess sonocatalytic activity and thus cannot generate bactericidal reactive species, compromising the stability and long‐term functionality of implants.^[^ ^15^ ^]^ Therefore, it is imperative to develop surface modification strategies to endow titanium‐based implants with ultrasound‐responsive antibacterial and osteoinductive capabilities. Among them, ultrasound sensitizers such as metal‐organic framework porphyrin‐based compounds, carbon‐based materials, and piezoelectric nanomaterials have demonstrated US‐responsive capabilities.^[^ ^16^, ^17^, ^18^, ^19^ ^]^ Piezoelectric nanomaterials, due to their non‐centrosymmetric crystal structure, enable them to utilize the mechanical effect of ultrasound to generate a piezoelectric potential, which induces a catalytic reaction and generates ROS.^[^ ^20^, ^21^, ^22^, ^23^, ^24^ ^]^ Many biologically‐safe inorganic piezoelectric materials, including molybdenum disulfide (MoS_2_),^[^ ^25^, ^26^, ^27^ ^]^ zinc oxide (ZnO),^[^ ^28^, ^29^ ^]^ bismuth ferrite (BiFeO_3_),^[^ ^30^, ^31^ ^]^ and barium titanate (BTO),^[^ ^32^, ^33^ ^]^ have been widely used in deep‐tissue antimicrobial therapy. However, the high complexation rate of electron‐hole pairs is still a major limiting factor for acoustic catalysis. Therefore, how to accelerate the ultrasonic piezoelectric charge transfer is the key to realizing efficient SDT.^[^ ^34^, ^35^ ^]^

BTO, a conventional piezoelectric material, has a high piezoelectric constant and a large electromechanical coupling coefficient, and is frequently used for deep bone infection treatment due to its excellent biocompatibility.^[^ ^36^, ^37^, ^38^ ^]^ The main catalytic process is that BTO deforms and undergoes a polarization reaction when excited by US, causing positive and negative charges to migrate to the two levels, forming a piezoelectric field that catalyzes the generation of ROS.^[^ ^39^ ^]^ Unmodified BTO, under ultrasonic excitation, exhibits low catalytic activity, so many studies are currently focused on developing BTO derivatives with high catalytic activity. Among them are the enhancement of the piezoelectric constant of BTO by creating oxygen defects, where the presence of oxygen vacancies can effectively adsorb and activate O_2_ on the surface of BTO, therefore enhancing the piezoelectric catalytic activity.^[^ ^40^, ^41^, ^42^ ^]^ The loading of active antibacterial factors triggers such as NO and CO, for a synergistic treatment of bacterial infections.^[^ ^43^ ^]^ Zhang et al. utilized NIR/US to assist in the combined antibiotic treatment of osteomyelitis.^[^ ^44^ ^]^ Li et al. prepared a BTO@Au structure with a Schottky heterojunction to generate ROS under ultrasound for skin antimicrobial therapy.^[^ ^45^ ^]^ And Wang et al. utilized the piezoelectricity of BTO in combination with thermotherapy to induce copper death in bacteria.^[^ ^46^ ^]^ Analyzing the previous works, we found that the lower carrier mobility is the main influencing factor for the low catalytic performance of BTO. On this basis, researchers have proposed various solutions, such as accelerating interfacial charge transport by creating heterojunctions can effectively solve this problem.

As a nonmagnetic semiconductor, the presence of unpaired electrons in TiO_2_ enables it to be excited by US to produce electron and hole pairs, which is often used in SDT. However, the wide band gap of TiO_2_ makes it difficult to excite its acoustic electrons; through defect engineering, not only can the band gap of TiO_2_ be adjusted, but also its intrinsic electronic spin state can be changed, which can significantly improve its sonocatalytic performance.^[^ ^47^ ^]^ For example, the spin‐polarized Fe‐Ti electrodes enriched with Ti^3+^ species designed by oxygen defect engineering can enhance the interaction between the Fe‐Ti electrode and key intermediates and improve the catalytic activity^[^ ^48^ ^]^; Mi et al. prepared TiO_2_ with asymmetric spin‐up and spin‐down states by manipulating the defects of titanium dioxide, which greatly enhances its catalytic activity.^[^ ^49^, ^50^, ^51^ ^]^ This is mainly due to the fact that the oxygen defects and Ti^3^⁺ species not only accelerate charge transport but also improve catalytic efficiency by enhancing oxygen.^[^ ^48^, ^51^ ^]^

**Figure 1:**
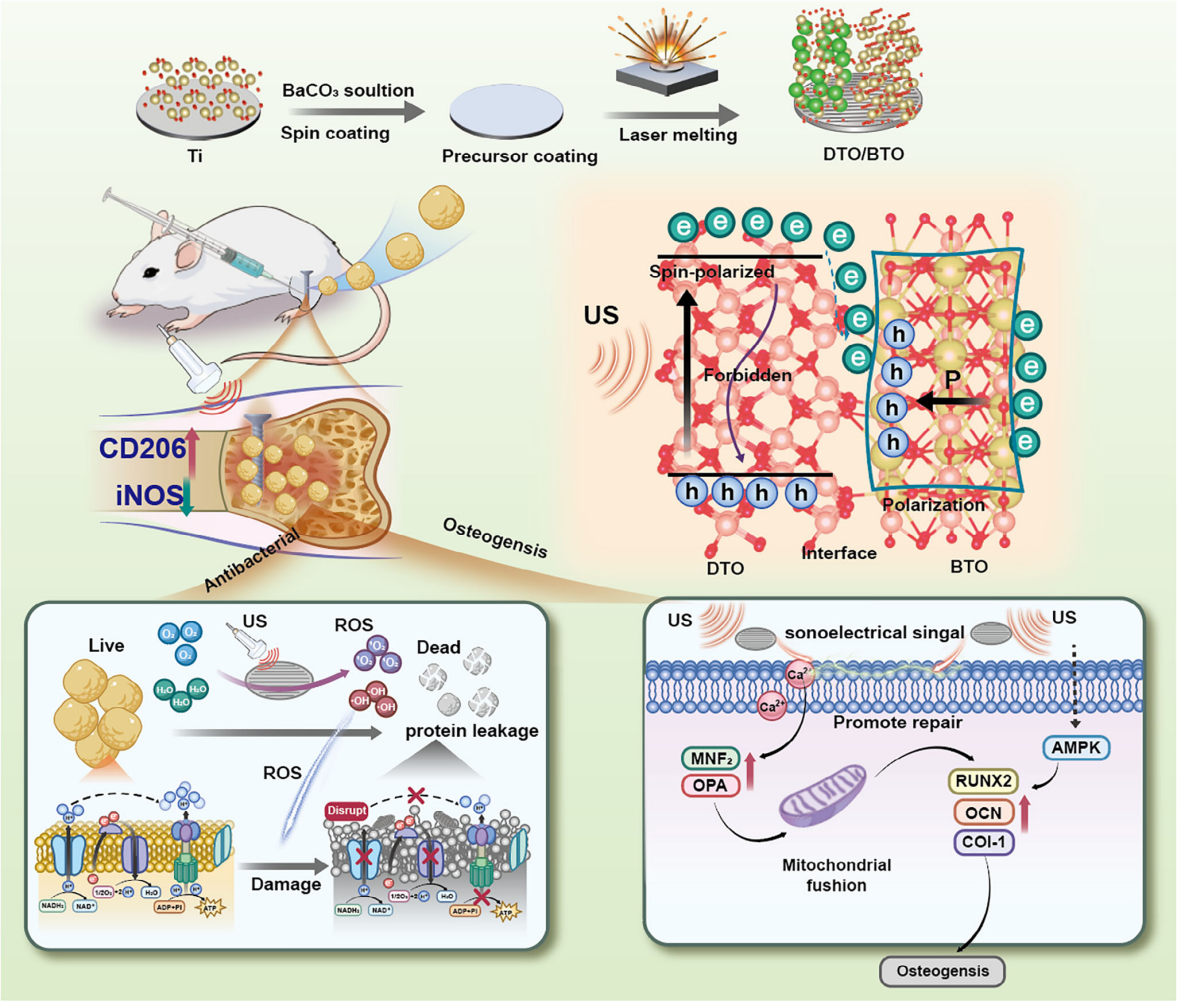
Schematic illustration of ultrasound‐active DTO/BTO for treating *S. aureus*‐infected bone defect and promoting osteogenic differentiation.

In this study, we prepared a defective titanium dioxide and barium titanate heterojunction enriched with Ti^3+^ species using the laser fusion technique under high temperature and rapid cooling conditions for the treatment of deep bone tissue infection under ultrasonic excitation. The introduction of oxygen vacancies narrowed the band gap of TiO_2_, allowing better generation of ultrasound electrons. Density Functional Theory (DFT) experiments have shown that the asymmetric spin states in DTO/BTO can accelerate the ultrasonic charge transfer and inhibit the complexation of electron and hole pairs, which improves the ROS yield to scavenge bacteria. In addition, as shown in **Figure** **1**, RNA sequencing results further revealed that microcurrents generated by ultrasound‐excited DTO/BTO were able to up‐regulate MFN2 and OPA genes to promote mitochondrial fusion, which in turn increased the expression levels of mitofusin 2 (RUNX2), osteocalcin (OCN), and collagen (COL‐1) genes, and ultimately promoted osteogenic differentiation. In vivo experiments demonstrated that DTO/BTO effectively treated osteomyelitis and induced the production of new bone around the implant and accelerated bone tissue repair, demonstrating the remarkable therapeutic potential of ultrasound‐assisted DTO/BTO.

## 2 Results and Discussion

### 2.1 Characterization of DTO/BTO

**Figure 2:**
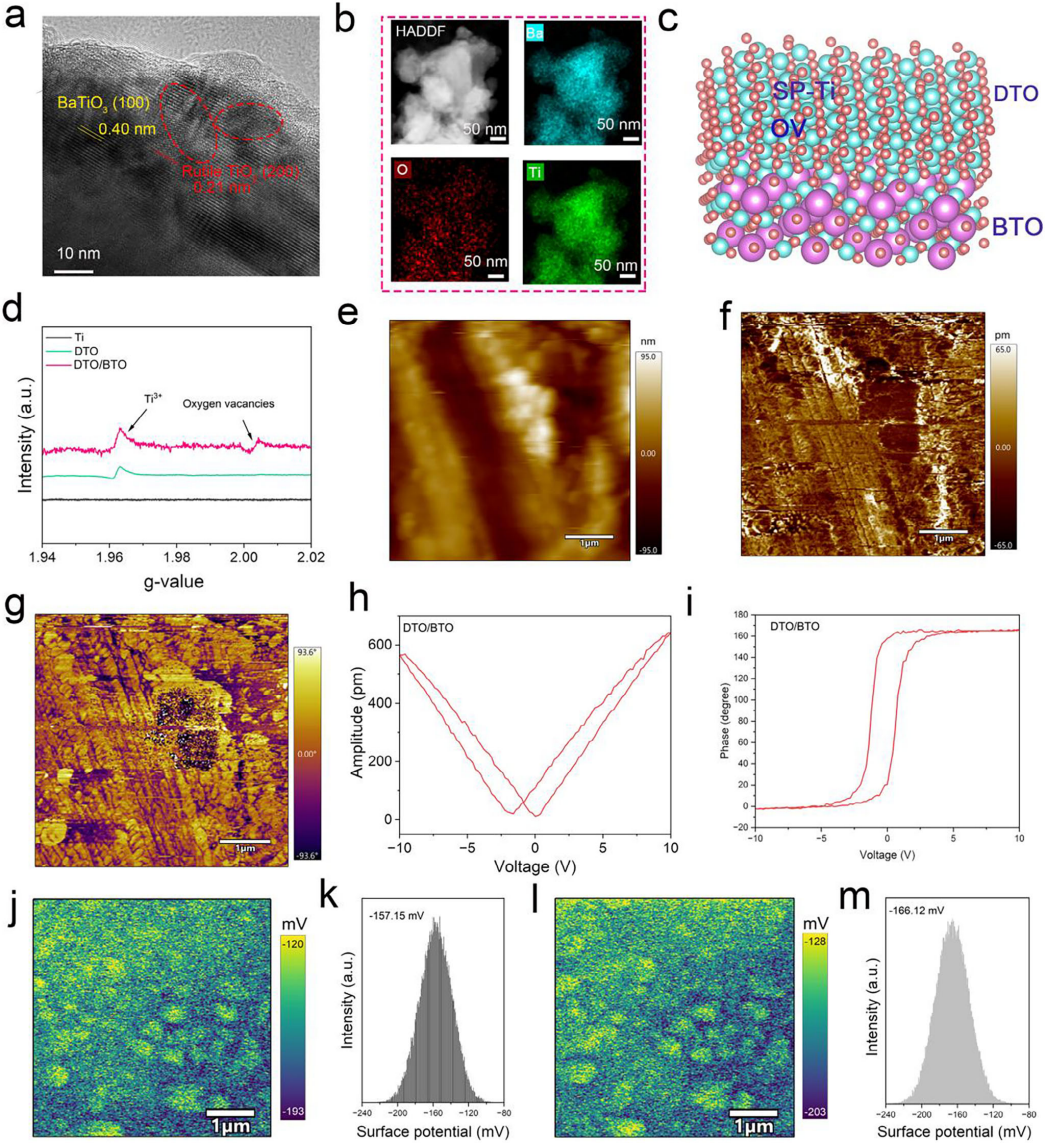
Characterization of DTO/BTO. a) HRTEM of DTO/BTO. b) Elemental mapping of DTO/BTO. c) The atomic structure diagram of DTO/BTO. d) EPR spectra of DTO and DTO/BTO, e) PFM morphology. f) amplitude image of DTO/BTO, and g) phase image. h) Piezoresponsive amplitude curve, and i) phase curve. j) KPFM image (scale bar = 1000 nm) and (k) corresponding surface potential distribution of DTO/BTO before ultrasound‐irradiated KPFM. l) KPFM image (scale bar = 1000 nm) and (m) corresponding surface potential distribution of DTO/BTO on ultrasound‐irradiated KPFM.

The DTO/BTO heterojunction coating was prepared using laser cladding. Briefly, the prepared BaCO_3_ solution was uniformly spin‐coated onto the polished Ti substrate. After the sample dried, the Ti surface was laser cladded, as shown schematically in Figure 1. The surface morphologies of different samples were observed using scanning electron microscopy (SEM) and are shown in Figure S1a–c (Supporting Information). Compared to the smooth surface of the pure titanium substrate, the laser‐treated Ti surface exhibits densely packed nanostructures with diameters ranging from 400–500 nm (Figure S1b, Supporting Information), whereas the laser‐treated BaCO_3_ material covers the titanium substrate with smaller structures, approximately 200–300 nm in diameter (Figure S1c, Supporting Information). As illustrated in Figure S2a (Supporting Information), the cross‐sectional view of DTO/BTO shows that a coating layer forms on the titanium surface after laser cladding, confirming the successful preparation of the coating. Further examination of the surface morphology of the DTO/BTO samples by high‐resolution transmission electron microscopy (HRTEM), as shown in the area highlighted in red in **Figure** **2**a, reveals a lattice spacing of 0.21 nm for TiO_2_, matching the (200) crystal plane, and a lattice spacing of 0.40 nm for BaTiO_3_, matching the (100) crystal plane. The lattice edge of TiO_2_ (200) also exhibits localized bending deformation (indicated by the red dashed circle) during the laser melting process.^[^ ^54^, ^55^, ^56^ ^]^ Furthermore, the elemental mapping shown in Figure 2b indicates a uniform distribution of Ba, Ti, and O elements on the surface of DTO/BTO, suggesting the successful creation of the DTO/BTO heterojunction with tightly connected interfaces through the laser cladding process. Figure 2c illustrates the atomic structure of the DTO/BTO heterojunction obtained by density functional theory (DFT).

X‐ray diffraction (XRD) to determine the crystalline structure of Ti, DTO, and DTO/BTO, as shown in Figure S3 (Supporting Information), reveals the presence of the typical peaks of Ti and anatase TiO_2_ in Ti, DTO, and DTO/BTO, which are attributed to spontaneously formed TiO_2_. Compared to pure Ti, new peaks at 27.4° (110), 36.2° (101), and 43.6° (210) appear in the XRD patterns of DTO and DTO/BTO, corresponding to rutile TiO_2_. This is attributed to the high temperatures generated during the laser cladding process, which convert anatase to rutile. We also observed a gradual shift in the diffraction peaks at 25.31° to a higher degree, which might correlate to the structural distortion induced by OVs and Ti^3+^. In contrast to DTO, distinct diffraction peaks appear for DTO/BTO at 22.1°, 31.4°, 39.0°, 45.1°, and 56.1° that correspond to the (100), (110), (111), (200), and (211) planes, respectively, of perovskite‐structured BaTiO_3_.^[^ ^57^, ^58^, ^59^ ^]^ As shown in Figure S4 (Supporting Information), the contact angle is lower for the laser‐clad DTO than for untreated pure Ti, indicating that DTO has a higher surface roughness. Additionally, compared to Ti and DTO, DTO/BTO exhibits a more hydrophilic surface. These results align well with the changes in the surface morphology of Ti, DTO, and DTO/BTO shown in Figure S1a–c (Supporting Information).

Further analysis of the surface chemical compositions of the different samples by X‐ray photoelectron spectroscopy (XPS) revealed that the survey spectra of DTO/BTO displayed Ba 3d, O 1s, Ti 2p, and C 1s peaks (Figure S5, Supporting Information). The high‐resolution XPS analysis of O 1s of Ti (Figure S6a, Supporting Information), DTO (Figure S7a, Supporting Information), and DTO/BTO was fitted to three peaks (Figure S8a, Supporting Information). The peak at 531.4 eV belongs to the O atom around the oxygen vacancy, while the peak at 529.5 eV is ascribed to the lattice oxygen. By contrast, the OVs in Ti are negligible, while the concentration of OVs is greater in DTO/BTO than in DTO.^[^ ^60^ ^]^ The peaks at a binding energy of 532.2 eV were attributed to O‐H, possibly formed due to the presence of ·OH groups or to H_2_O absorbed on the surface. In the Ti 2p XPS spectra, Ti exhibited two peaks at 458.6 eV (Ti 2p_3/2_) and 464.6 eV (Ti 2p_1/2_), attributed to Ti^4+^ in TiO_2_ (Figure S6b, Supporting Information).^[^ ^54^, ^57^ ^]^ As shown in Figures S7b and S8b (Supporting Information), DTO and DTO/BTO showed two peaks at 459.0 eV (Ti 2p_3/2_) and 465.9 eV (Ti 2p_1/2_), corresponding to Ti^4+^ in TiO_2_. Additionally, peaks at 457.8 and 463.8 eV are observed in DTO/BTO, attributed to Ti^3+^ near the oxygen vacancy sites. Compared to DTO, DTO/BTO contains more Ti^3+^ species.^[^ ^56^ ^]^ The Ba 3d high‐resolution spectrum was fitted with peaks at 795.3 and 779.9 eV, corresponding to Ba^2+^ in the perovskite structure (Figure S8c, Supporting Information).^[^ ^23^, ^39^, ^58^, ^61^ ^]^ These results further confirm that the phase composition of DTO/BTO is BaTiO_3_. Subsequent electron spin resonance (ESR) spectroscopy, employed to identify Ti^3+^ species and OVs (Figure 2d), demonstrated the successful generation of abundant Ti^3+^ species in both DTO/BTO and DTO coatings, with a higher concentration of OVs and Ti^3+^ species in DTO/BTO than in DTO.^[^ ^48^, ^62^ ^]^

Our utilization of the inverse piezoelectric effect of piezoresponse force microscopy (PFM) confirmed the piezoresponse properties of DTO/BTO. As shown in Figure 2e, the high‐magnification surface morphology of DTO/BTO reveals irregular nanoparticles. Figure 2f illustrates the piezoresponse signal amplitude of DTO/BTO, while Figure 2g displays its phase image. The PFM results indicate the presence of antiparallel ferroelectric domain structures in DTO/BTO. As illustrated in Figure 2h,i, the PFM amplitude response exhibits a characteristic butterfly curve, and the corresponding PFM phase demonstrates polarization reversal. When a voltage is applied, significant changes occur in the polarization state in certain regions, and substantial polarization intensity is maintained upon removal of the voltage (Figure S9, Supporting Information). These findings suggest that DTO/BTO possesses piezoelectric response characteristics and can generate polarization under external pressure, thereby facilitating piezoelectric catalysis.^[^ ^12^, ^30^, ^33^, ^44^ ^]^ In this study, in‐situ ultrasonic excitation Kelvin probe force microscopy (KPFM) was employed to investigate the surface charge effects of DTO/BTO, with a focus on observing the distribution of electron‐hole pairs generated by ultrasonic stimulation. As shown in Figure 2j, the surface potential of DTO/BTO was measured to be −157.15 mV in the absence of ultrasonic stimulation. Under ultrasonic excitation, the average surface potential of DTO/BTO decreased to −166.12 mV (Figure 2l,m).^[^ ^64^, ^65^ ^]^ This reduction in the average surface potential under ultrasonic stimulation indicates that the acoustically excited electrons were transferred to the surface of DTO/BTO, leading to electron accumulation.

### 2.2 Sonocatalytic Performance of DTO/BTO

**Figure 3:**
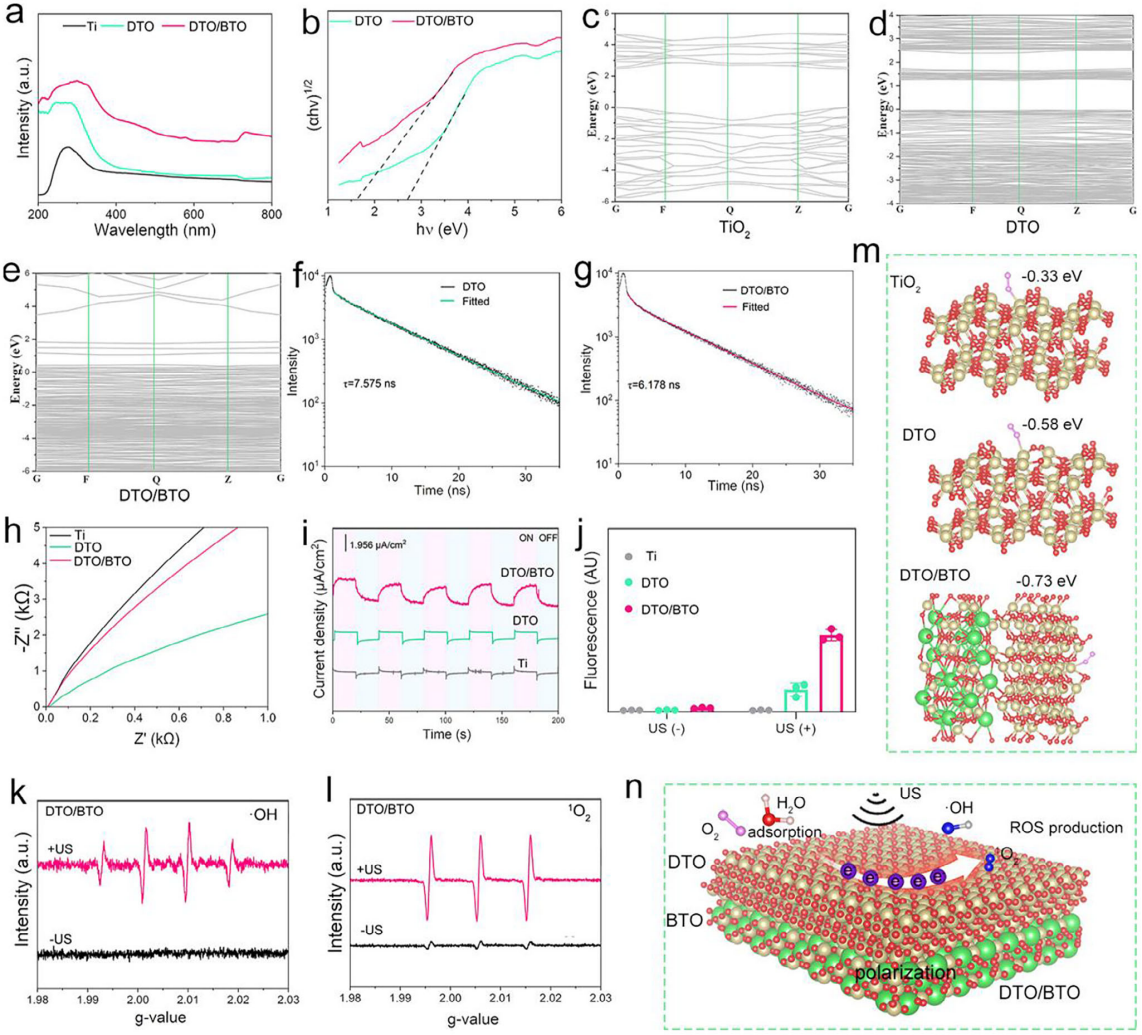
The generation of ROS and sonocatalytic performance of DTO/BTO under US. a) the UV–vis absorption of DTO/BTO. b) the band structure calculated by UV–vis absorption spectrum of DTO/BTO. The band structure of c) TiO_2_, d) DTO, and e) DTO/BTO calculated by DFT. Time‐resolved photoluminescence (TRPL) spectra of f) DTO, and g) DTO/BTO, h) EIS test of different samples. i) Ultrasonic current test of different samples. j) ROS production with DCFH fluorescence probe under US irradiation for 20 min of different samples. k) ESR spectra of ·OH. l) ESR spectra of ^1^O_2_. m) oxygen adsorption location, and oxygen adsorption energy of different samples. n) A schematic diagram of fast interfacial charge separation at the DTO/BTO heterojunction.

The band structure of DTO/BTO affects the separation efficiency of electron‐hole pairs under ultrasonic excitation, thereby influencing the system's ultrasonic performance. In this study, the bandgap of different materials was calculated using ultraviolet visible‐near infrared (UV‐Vis‐NIR) absorption spectra. As shown in **Figure** **3**a, DTO/BTO exhibits stronger light absorption compared to Ti and DTO. As shown in Figure 3b, calculations reveal that the band gaps of DTO and DTO/BTO are 2.71 and 1.68 eV, respectively. The band structure of the DTO/BTO material is further investigated using DFT calculations. As shown in Figure 3c‐e, the band gap diagram of TiO_2_ and DTO shows that the band gap of DTO is narrower than TiO_2_ due to the introduction of OVs and titanium vacancy. By contrast, DTO/BTO has the narrowest bandgap compared to TiO_2_ and DTO, which corresponds to the trend of bandgap reduction calculated by UV‐Vis‐NIR absorption spectra. The narrower band gap favors the generation of electrons during sonication.

The carrier mobility of DTO/BTO, investigated using time‐dependent PL fluorescence spectroscopy (TRPL), showed a lower fluorescence lifetime of 6.178 ns for DTO/BTO compared to 7.575 ns for DTO (Figure 3f,g). This difference is attributed to the migration of carriers in the heterointerface when a heterojunction forms between DTO and BTO, resulting in a lower fluorescence lifetime. Further examination of the piezoelectric catalytic performance of DTO/BTO using an electrochemical workstation generated electrochemical impedance spectroscopy (EIS) plots of the different samples under ultrasonic irradiation, as shown in Figure 3h. The EIS pattern of Ti exhibits the largest semicircle, indicating the largest impedance during charge transfer, whereas the charge transfer resistance is lower for DTO than for Ti, suggesting that the formation of oxygen vacancies and defect‐enrich Ti^3+^ promotes charge transfer. Conversely, the EIS patterns of Ti and DTO/BTO exhibit the smallest semicircles compared to Ti, indicating that heterojunction formation further reduces the impedance for charge transfer.

Figure 3i shows the ultrasonic current density plot of each group investigated using the electrochemical workstation. Under ultrasonic excitation, the current density was lower for the pure titanium group, indicating the poor ability of Ti to generate US‐excited electrons. The higher ultrasonic current density for DTO than for Ti indicates that the oxygen defects formed in DTO after laser treatment are favorable for current generation. This is attributed to the fact that defect‐rich Ti^3^⁺ species can transfer ultrasound‐excited electrons, thereby facilitating the separation of electron‐hole pairs. Meanwhile, compared with Ti and DTO, DTO/BTO exhibits the largest ultrasonic current density, suggesting that the formation of heterojunction greatly accelerates the generation of acoustically excited electrons and provides favorable conditions for the generation of ROS.

The generation of reactive oxygen species (ROS) by different materials, both with and without ultrasound, was measured using the DCFH fluorescence method (Figure 3j). In the absence of ultrasound excitation, each group produced negligible ROS. However, after 20 min of ultrasound treatment, the DTO/BTO group exhibited the highest ROS production, followed by the DTO group, whereas the Ti group generated a comparatively small amount of ROS, again suggesting that the heterojunction formed by DTO and BTO significantly enhances ROS generation. ROS production by DTO/BTO was further examined using electron spin resonance (ESR) spectroscopy to explore hydroxyl radical (·OH) production in DTO/BTO using 5,5‐dimethyl‐1‐pyrroline N‐oxide (DMPO) as a trapping agent. As shown in Figure 3k, no signal appeared in the DMPO‐treated DTO without ultrasonic excitation. However, after ultrasonic treatment, the appearance of quadruple peaks in the plots indicated that the ultrasonic excitation of DTO/BTO produces hydroxyl radicals. As shown in Figure 3l, using 2,2,6,6‐tetramethylpiperidine (TEMP) as the capture agent revealed single‐line state oxygen generation by DTO/BTO. The signal intensity was stronger when generated under ultrasonic excitation than without ultrasound treatment, indicating that ultrasonic treatment of DTO/BTO enhances the generation of single‐line state oxygen. Figure 3m shows the sites of O_2_ adsorption on the surface of different materials and the corresponding oxygen adsorption energies. For TiO_2_, the adsorption sites for O_2_ are mainly concentrated on Ti^4+^, with an adsorption energy of −0.33 eV. In comparison, owing to the abundance of Ti^3+^ species in DTO, oxygen is adsorbed onto the Ti^3+^ sites surrounding the oxygen vacancies (OVs). Density functional theory (DFT) calculations indicate that the adsorption of DTO energy is −0.58 eV, whereas the oxygen adsorption energy of DTO/BTO is −0.73 eV. Therefore, when a heterojunction interface is formed, the system is more likely to adsorb oxygen.

### 2.3 The Mechanism of Sonocatalytic Performance

First‐principles calculations were used to study the impact of spin polarization effects on charge transfer. The primary focus was on how spin polarization affects carrier transport and electron transfer. Additionally, ESR results show that the reactive oxygen species (ROS) generated by DTO/BTO under ultrasound (US) excitation are singlet oxygen (^1^O_2_) and hydroxyl radicals (·OH), indicating that oxygen activation plays a crucial role in ROS generation. Therefore, we also investigated the effect of DTO/BTO on the transition of O_2_ from the initial state (IS, gaseous O_2_) to the transition state (TS). The schematic diagram of the DTO/BTO ultrasound catalytic mechanism is shown in Figure 3n.

**Figure 4:**
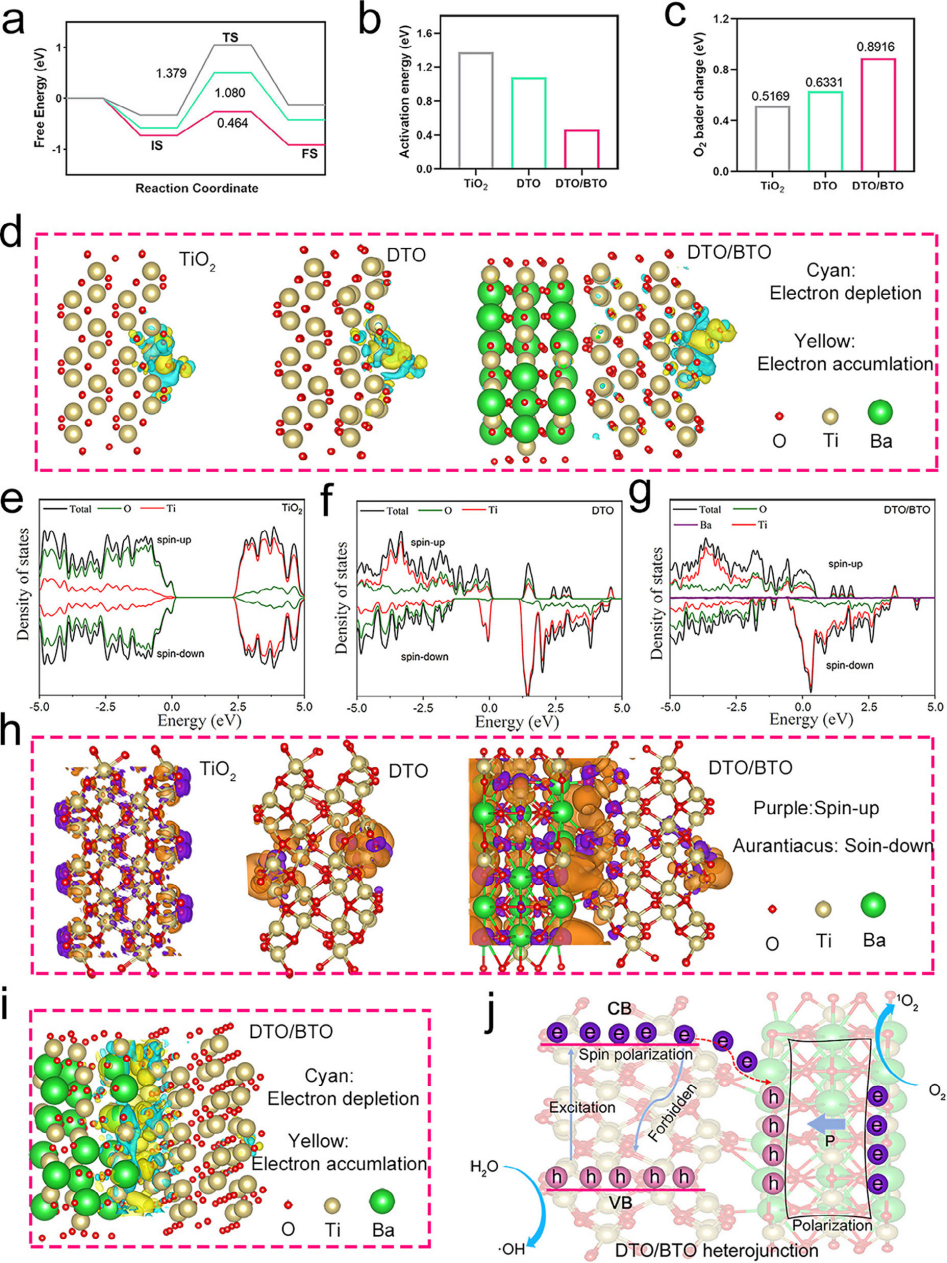
DFT calculations to investigate the rapid mechanism of interfacial electron transfer of DTO/BTO. a) Gibbs free energy diagram of O_2_ activation. IS, TS, and FS correspond to the initial state, transition state, and final state, respectively. b) O_2_ activation energy of different samples, c) O_2_ barde charge, d) O_2_ charge density difference of different samples. Density of state (DOS) of e) TiO_2_, f) DTO, g) DTO/BTO. h) Spin density of DTO/BTO heterojunction (purple and brown shells denote spin‐up and spin‐down components, respectively; the isosurface is set to 0.01 e Å3), i) charge density difference of DTO/BTO. j) mechanism diagram of enhanced ROS generation by DDTO/BTO under US treatment.

**Figure** **4**a shows the optimized reaction pathway and corresponding Gibbs free energy of O_2_ on the DTO/BTO surface, calculated using DFT. The activation energies for TiO_2_, DTO, and DTO/BTO were 1.379, 1.080, and 0.464 eV, respectively (Figure 4b), indicating that oxygen vacancies and the formation of heterojunction interfaces facilitate the activation of O_2_. Meanwhile, in the final state (FS), the Gibbs free energy is significantly lower for DTO/BTO than for TiO_2_, indicating a more reliable O_2_ activation reaction on the DTO/BTO surface. We further elucidated the impact of oxygen on electron transfer by examining the variations in the charge density surrounding the oxygen atoms. As shown in Figure 4c, the Bader charges of O_2_ in various samples were calculated to be 0.5169 eV for TiO_2_, 0.6331 eV for DTO, and 0.8916 eV for DTO/BTO. This finding indicates that the electrons generated on Ti^3+^ in DTO/BTO are more likely to migrate to O_2_, further accelerating the charge transfer rate (Figure 4d). We also systematically examined the spin properties of Ti^3+^ in DTO, as these significantly influence carrier transport. As shown in Figure 4e–g, the density of states (DOS) near the Fermi level for the spin‐up and spin‐down states in TiO_2_ are similar, indicating negligible electron polarization. By contrast, the defect‐rich Ti^3+^ of Ti 3d in DTO leads to notable electron polarization, with the DOS for spin‐up states lower than for spin‐down states. Moreover, as shown in Figure 4g, the DOS is much lower for spin‐up states near the Fermi level in DTO/BTO is much lower than that for spin‐down states, resulting in pronounced electron polarization, which can provide more spin‐down electrons during the catalytic process.

The 3D spatial distribution of spin polarization (Figure 4h) shows that DTO/BTO exhibits extensive negative spatial spin polarization (SSP) within the unit cell, indicating a low likelihood of spin polarization reversal in real space. However, the spin polarization at the Fermi level is much lower for TiO_2_ and DTO than for DTO/BTO. These findings confirm the presence of spin polarization in the defect‐rich and Ti^3+^ DTO/BTO heterojunction, where the parallel alignment of electron spin polarization can further enhance charge separation and surface reaction efficiency during sonocatalysis. Figure 4i shows the differential charge distribution of DTO/BTO, where electron accumulation occurs on the BaTiO_3_ side (yellow), and electron depletion is observed on the TiO_2_ side. This indicates that the establishment of interface engineering is more conducive to electron migration.

The catalytic mechanism of DTO/BTO under ultrasonic excitation is illustrated in Figure 4j. Due to its piezoelectric properties, BTO undergoes polarization upon ultrasonic excitation, leading to charge separation. Defective titanium dioxide acts as a sonosensitizer, generating electrons upon ultrasonic excitation. Under the influence of spin polarization, the recombination of electron‐hole pairs is suppressed. The built‐in electric field generated when BTO comes into contact with DTO further promotes the separation of electrons and holes, resulting in the production of a significant amount of ROS.^[^ ^19^, ^23^ ^]^

### 2.4 In Vitro Sonodynamic Antibacterial Performance

**Figure 5:**
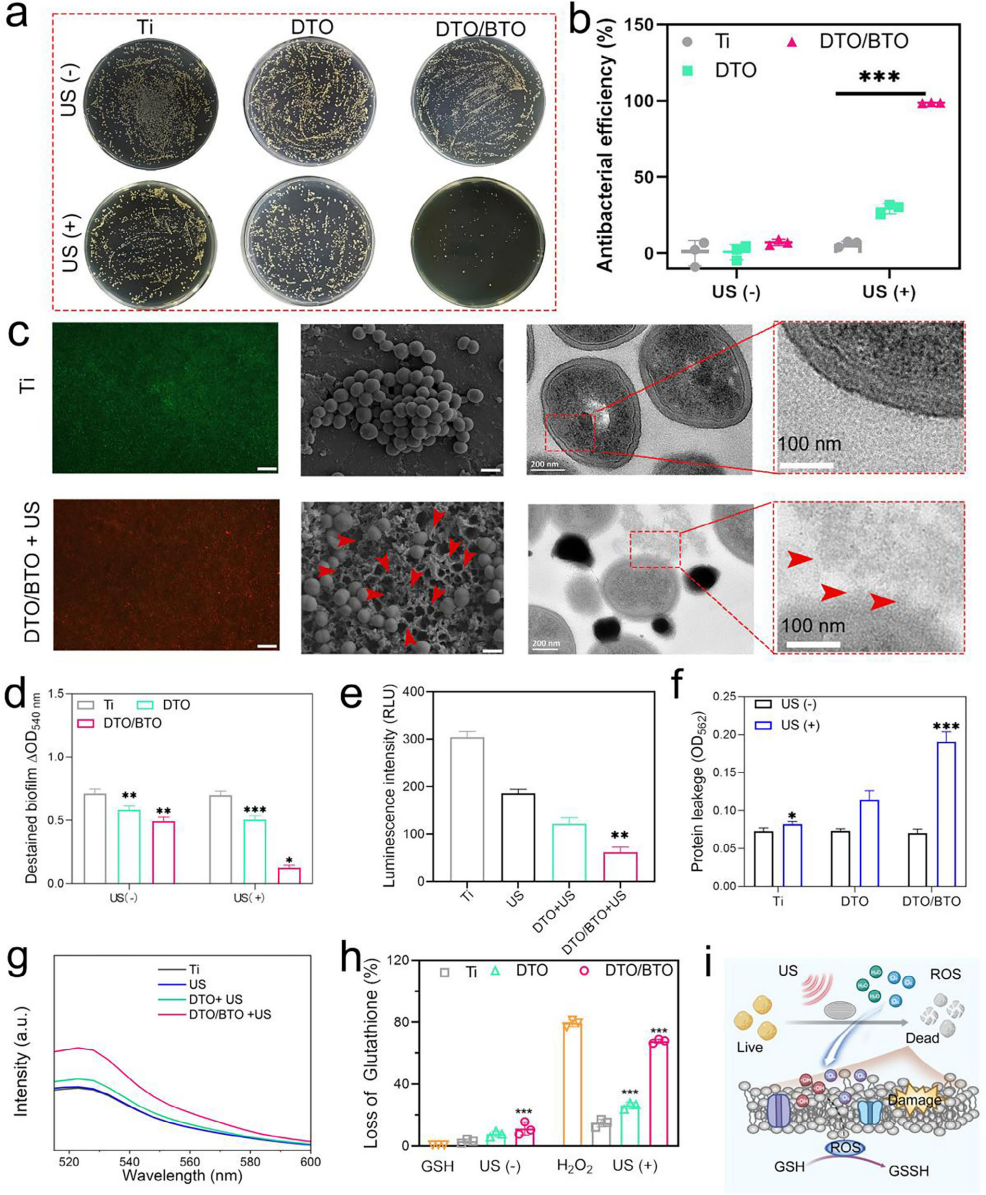
In vitro antibacterial performance of DTO/BTO. a) The spread plate images of S. aureus after treatments with Ti – US, DTO – US, DTO/BTO – US, Ti + US, DTO + US, and DTO/BTO + US. b) Number of S. aureus colonies after different treatments. c) Fluorescent images of living (green) and dead (red) staining bacterial after treatment by different samples (Scale bars: 50 µm), the SEM images of morphologies of S. aureus on different samples, and the TEM images of S. aureus treatments with Ti, Ti + US, DTO + US, and DTO/BTO + US. d) OD value of crystal violet solution after staining the biofilms formed cultured on the samples (The error bars indicate means ± SD, *n* = 3. *p < 0.05, **p < 0.01, ***p < 0.001). e) ATP detection of the S. aureus treated by samples. (The error bars indicate means ± SD, *n* = 3. *p < 0.05, **p < 0.01, ***p < 0.001). f) Detection of protein leakage of S. aureus treatments with different groups through bicinchoninic acid assay (BCA). (The error bars indicate means ± SD, *n* = 3. *p < 0.05, **p < 0.01, ***p < 0.001). g) The fluorescence intensity of *S. aureus* after treatments with Ti, Ti + US, DTO + US, and DTO/BTO + US characterized by DCFH‐DA probe. (The error bars indicate means ± SD, n = 3. *p < 0.05, **p < 0.01, ***p < 0.001). h) Glutathione (GSH) deletion ability of different groups. (The error bars indicate means ± SD, *n* = 3. *p < 0.05, **p < 0.01, ***p < 0.001). i) The schematic diagram of antibacterial mechanism of DTO/BTO under ultrasonic stimulation. (The error bars indicate means ± SD, n = 3. *p < 0.05, **p < 0.01, ***p < 0.001).

A spread plate and the corresponding number of colonies for *S. aureus* were used to assess the antibacterial performance of DTO/BTO. **Figure** **5**a shows the bacterial colonies following US irradiation for 20 min and without US irradiation. Bacterial colonies in the Ti and DTO groups were similar without US irradiation, indicating a negligible antimicrobial effect by either surface, whereas the DTO/BTO surface showed a weak antimicrobial effect (8.36%) (Figure 5b). Under US excitation, only a small number of colonies were detected in the DTO/BTO group, and the calculated antimicrobial rate reached 99.83%, whereas in the other groups, more bacteria were still present, although the antimicrobial rate was significantly higher for the DTO group than for the Ti group. Compared to the Ti group without US irradiation, the antibacterial rate was 77.81% for the Ti group under ultrasonic excitation, which was attributed to the effect of ultrasonic heat. The antibacterial performance of the material was tested using only one antibacterial cycle, but increasing the number of antibacterial cycles enhanced the antibacterial rate. Therefore, DTO/BTO could completely kill bacteria in two to three cycles, indicating the promising potential of the coating for antibacterial applications. Figure 5c shows the results for bacterial fluorescence staining and the morphology of *S. aureus*. Live bacteria were stained green by the fluorescent dye, while dead bacteria were stained red. The bacterial fluorescence in the Ti group and after US treatment appeared green (Figure S10a, Supporting Information), indicating that most bacteria were alive. However, after 20 min of ultrasound irradiation, the DTO/BTO group showed a significant number of dead bacteria (red fluorescence), consistent with the plating results, indicating that the DTO/BTO material possesses good antibacterial properties under ultrasound irradiation.

The morphology of treated *S. aureus* samples observed by SEM (Figure 5c) (the middle image) showed that the bacteria in the Ti group and the US group displayed morphological integrity. The bacterial morphology in the DTO + US group displayed slight shrinkage compared to normal bacteria (Figure S10b, Supporting Information). The red arrows mark the damaged parts of the bacteria, and the DTO/BTO group showed severely deformed and damaged bacteria under US irradiation. TEM images of the bacteria, shown in Figure 5c (the image on the far right) and Figure S11 (Supporting Information), reveal complete bacterial walls of *S. aureus* in the Ti and US groups, whereas the bacterial membranes were somewhat disrupted in the DTO + US group (shown by red arrows) and severely disrupted in the DTO/BTO + US group, in agreement with the antibacterial results shown in Figure 5a.

The excellent antibacterial effect of DTO/BTO led us to further investigate the effectiveness of these samples in removing bacterial biofilms formed on the implant surface under different conditions. The crystal violet assay was employed to evaluate biofilm growth on DTO/BTO after 2 days of incubation. As shown in Figure 5d and Figure S12 (Supporting Information), following 20 min of ultrasonic irradiation, the biofilm removal rate was 82.3% for the DTO/BTO group compared to the Ti group, indicating that DTO/BTO exhibits significant biofilm resistance.

One possible explanation for the disruption of biofilm formation is the disruption of ATP formation by infective bacteria. ATP serves as a vital energy molecule that is crucial for bacterial growth and various physiological activities, such as bacterial respiration. Upon bacterial death, ATP levels typically decrease.^[^ ^63^ ^]^ Further investigation of the ATP activity of *S. aureus* following different treatments revealed that bacteria subjected to US DTO/BTO treatment for 20 min had significantly lower ATP levels compared to the other groups (Figure 5e). This finding suggested that DTO/BTO+US effectively disrupts normal bacterial respiration, thereby impacting bacterial activity.

The antimicrobial mechanism of DTO/BTO was further investigated, given its excellent antimicrobial effect under US irradiation. As shown in Figure 5f, the protein leakage from *S. aureus* showed little change in the Ti, DTO, and DTO/BTO groups without US treatment. After ultrasonication, however, the protein leakage was negligible in the Ti group, but it increased significantly in the DTO and DTO/BTO groups, with the DTO/BTO group having the highest protein leakage. We also examined oxidative stress, as this is an important factor that inactivates bacteria. The intracellular ROS changes in bacteria were detected by 2′‐7′dichlorofluorescin diacetate (DCFH‐DA) probe (DCFH) (stronger fluorescence intensity indicates a greater level of intracellular ROS). The fluorescence graph shown in Figure S13 (Supporting Information) reveals the strongest fluorescence intensity (the highest absorption intensity at 525 nm) in the DTO/BTO+US‐treated bacteria (Figure 5g; Figure S13, Supporting Information), indicating that acoustic catalysis has a significant effect on the metabolic state of the bacteria. Examination of the levels of the endogenous antioxidant GSH was further used to assess oxidative stress within bacteria. As shown in Figure 5h, the group without US irradiation showed almost no GSH consumption, whereas the DTO/BTO+US group exhibited the greatest GSH depletion caused by reaction with the ROS produced by DTO/BTO under sonication excitation. This GSH depletion would reduce bacterial activity to a great extent, thereby promoting the removal of bacteria. The proposed antibacterial mechanism is shown in Figure 5i. The ROS generated by ultrasound‐activated DTO/BTO alters the permeability of the bacterial membrane, leading to the rupture of the bacterial cell membrane and the leakage of intracellular proteins. Under ultrasonic stimulation, the electrons on the bacterial cell membrane become disordered, thereby disrupting the bacterial respiratory chain. Additionally, ROS penetrates the bacteria and participates in the oxidation of glutathione, inducing oxidative stress and further reducing bacterial activity.

### 2.5 In Vitro Biocompatibility and Differentiation of Osteoblasts

**Figure 6:**
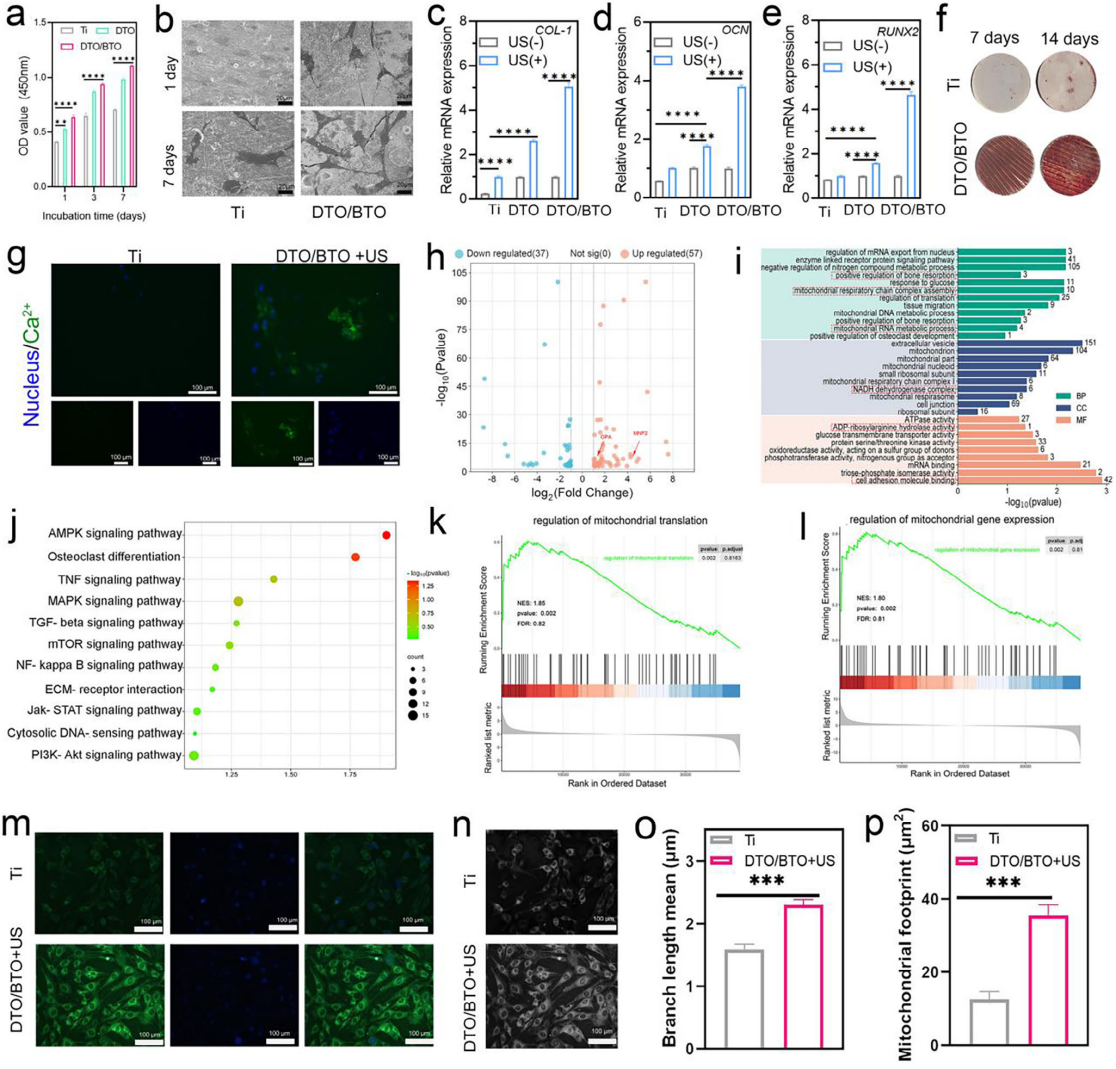
Biological safety and osteogenic performance. a) CCK8 test of the MSCs treat with different samples. (The error bars indicate means ± SD, *n* = 3. *p < 0.05, **p < 0.01, ***p < 0.001). b) SEM morphologies of MSCs cocultured with Ti and DTO/BTO. c‐e) OCN, RUNX2, and COL‐1 qRT‐PCR results of MCSs treated with Ti, DTO, DTO/BTO, with or without US irradiation. (The error bars indicate means ± SD, *n* = 3. *p < 0.05, **p < 0.01, ***p < 0.001). f) ARS staining images of Ti and DTO/BTO treated MCSs in different culture conditions. g) Fluorescent images of Ca^2+^ of US treated BMCSs cultured with Ti and DTO/BTO +US. h) Volcano plot of the transcriptomic analysis of the differential genes between the Ti and DTO/BTO + US groups. i) GO analysis of the DEGs after DTO/BTO + US treatment in MSCs. j) Bubble map of the KEGG enrichment analysis. k) regulation of mitochondrial translation, and l) regulation of mitochondrial gene expression based on GSEA. m) The morphological changes in the mitochondrial network were observed in MSCs using laser confocal microscopy (MitoTracker Green). n) Mitochondrial skeleton images obtained through Fiji software (upper plot), and o) statistical analysis of mitochondrial length, p) the mitochondrial footprint (bottom plot) by the Kruskal‒Wallis test (*n*≥3). (The error bars indicate means ± SD, 3. *p < 0.05, **p < 0.01, ***p < 0.001).

The biosafety of the different groups was assessed using the CCK8 assay as a way to ensure that the materials did not exhibit cytotoxic effects. As shown in **Figure** **6**a, cell viability was measured after co‐culturing the materials with cells for 1, 3, and 7 days. Compared to the Ti group, both the DTO and DTO/BTO groups showed an increasing trend in cell viability, with the highest survival rate observed in the DTO/BTO group after seven days. The live/dead fluorescent staining of BMSCs cultured on Ti, DTO, and DTO/BTO for 7 days, as shown in Figure S14 (Supporting Information), revealed predominantly green‐stained images in the different groups, confirming that the materials did not exhibit cytotoxicity.

As shown in Figure S15 (Supporting Information), the spread of MSCs on the different groups of materials, observed using FITC and DAPI staining, revealed better spreading of cells on DTO/BTO than on Ti or DTO. In addition, the morphology of cells on the surfaces of the different groups revealed by SEM images showed that the cells had a tendency to stretch outward, and filamentous tentacles appeared at the cell edges in the DTO/BTO group but not in the other Ti groups (Figure 6b). The reason for these alterations may be that the micro‐ and nanostructures on DTO/BTO provide growth sites for cells and promote cell proliferation and adhesion, which would provide strong conditions for osteogenic differentiation and extracellular matrix (ECM) mineralization.

Figure 6c–e shows the relative expression of osteogenic gene mRNAs. The transcript levels of the osteogenic genes COL‐1, OCN, and Runx2 were determined by qRT‐PCR. Runx2 is an important transcription factor regulating the expression of various osteogenic genes, and COL‐1, a fibrillar collagen, is also very heavily expressed during bone growth.^[^ ^12^ ^]^ After day 14, the expression levels of OCN, Runx2, and COL‐1 genes were higher in the DTO/BTO group than in all the other groups. These results demonstrated that DTO/BTO stimulated by ultrasound induced the expression of osteogenic genes. Furthermore, we tested whether DTO/BTO promotes osteogenic differentiation of cells by analyzing the mineralization of the ECM in the DTO/BTO group by staining cells cocultured for 7 days and 14 days with alizarin red. As shown in Figure 6f, the calcium nodulation after 14 days was significantly better in the DTO/BTO cells than in the Ti and DTO/BTO cells in the dark.

As shown in Figure 6g, the Ca^2+^ concentration was significantly higher in the US‐stimulated DTO/BTO group than in the Ti group, a combined with the ultrasound current density data shown in Figure 3i, this suggests that the intensity of the electrical signal is positively correlated with an increase in intracellular Ca^2+^ concentration, and the increase of calcium ion concentration can improve the differentiation of osteoblasts.^[^ ^22^ ^]^

In summary, the piezoelectric‐coated DTO/BTO prepared by laser solubility technology can induce cell proliferation and adhesion, while also promoting ECM mineralization using surface‐coated micro‐nanojunctions. Moreover, the piezoelectric signal generated by ultrasonic stimulation of DTO/BTO can promote the mRNA expression of osteogenic genes to promote osteogenic differentiation while also playing a role as a downstream second messenger to guide osteogenic differentiation by increasing the intracellular Ca^2+^ concentration.

### 2.6 RNA Sequencing for Osteogenesis Mechanism Exploration of MSCs

The mechanism that enhanced osteogenesis by DTO/BTO under US irradiation was examined using RNA sequencing. As shown in Figure S16 (Supporting Information), the replicates of three independent experiments were consistent. The differentially expressed genes (DEGs) identified included 37 downregulated genes and 57 upregulated genes when comparing the DTO/BTO + US group with the Ti group (Figure 6h). Further analysis revealed upregulation of the mitofusion‐2 (MFN2) and OPA genes, which play crucial roles in mitochondrial fusion. Mitochondria are essential organelles for metabolism and energy supply in eukaryotic cells; therefore, the upregulation of MFN2 and OPA gene levels would be expected to increase mitochondrial fusion, thereby promoting mitochondrial osteogenic differentiation.^[^ ^66^ ^]^ Figure S17 (Supporting Information) shows the differentially expressed genes highlighted in the heatmap, while Figure 6i shows the gene ontology (GO) enrichment analysis results, indicating that DTO/BTO+US promoted cellular biological processes, including mitochondrial respiratory chain complex assembly and mitochondrial RNA metabolic processes. The DTO/BTO+US treatment promoted upregulation of the generation of mitochondrial respiratory chain complex I and glucose transmembrane transporter activity. As shown in Figure 6j, the Kyoto Encyclopedia of Genes and Genomes (KEGG) enrichment analysis revealed upregulation of the AMPK pathway in BMSCs treated with DTO/BTO+US. Gene set enrichment analysis (GSEA) results further validated the enrichment of DEGs involved in the regulation of mitochondrial translation (Figure 6k), the regulation of mitochondrial gene expression (Figure 6l), regulation of the mitochondrial respiratory transport chain (Figure S18, Supporting Information), and biomineralization (Figure S19, Supporting Information).^[^ ^67^ ^]^

These results indicate that DTO/BTO+US can promote osteogenic differentiation by influencing mitochondrial fusion. We further explored the underlying mechanism using MitoTracker staining to examine changes in the mitochondrial network in BMSCs (Figure 6m). Following DTO/BTO+US treatment, the majority of MSCs mitochondria exhibited a dense reticular structure and displayed intense high‐density fluorescence (Figure 6n). These mitochondria also had a greater average branch length and occupied a larger area (Figure 6o). By contrast, mitochondria in the Ti group predominantly displayed a punctate morphology, with shorter lengths and smaller areas compared to those in the DTO/BTO+US group (Figure 6p). These findings suggest that DTO/BTO+US modulates mitochondrial dynamics in BMSCs, specifically enhancing mitochondrial fusion.^[^ ^68^ ^]^ The mechanism of osteoblast differentiation of DTO/BTO+US is shown in Figure 1.

### 2.7 In Vivo Antibacterial Therapy and Enhanced Osteogenic Differentiation

**Figure 7:**
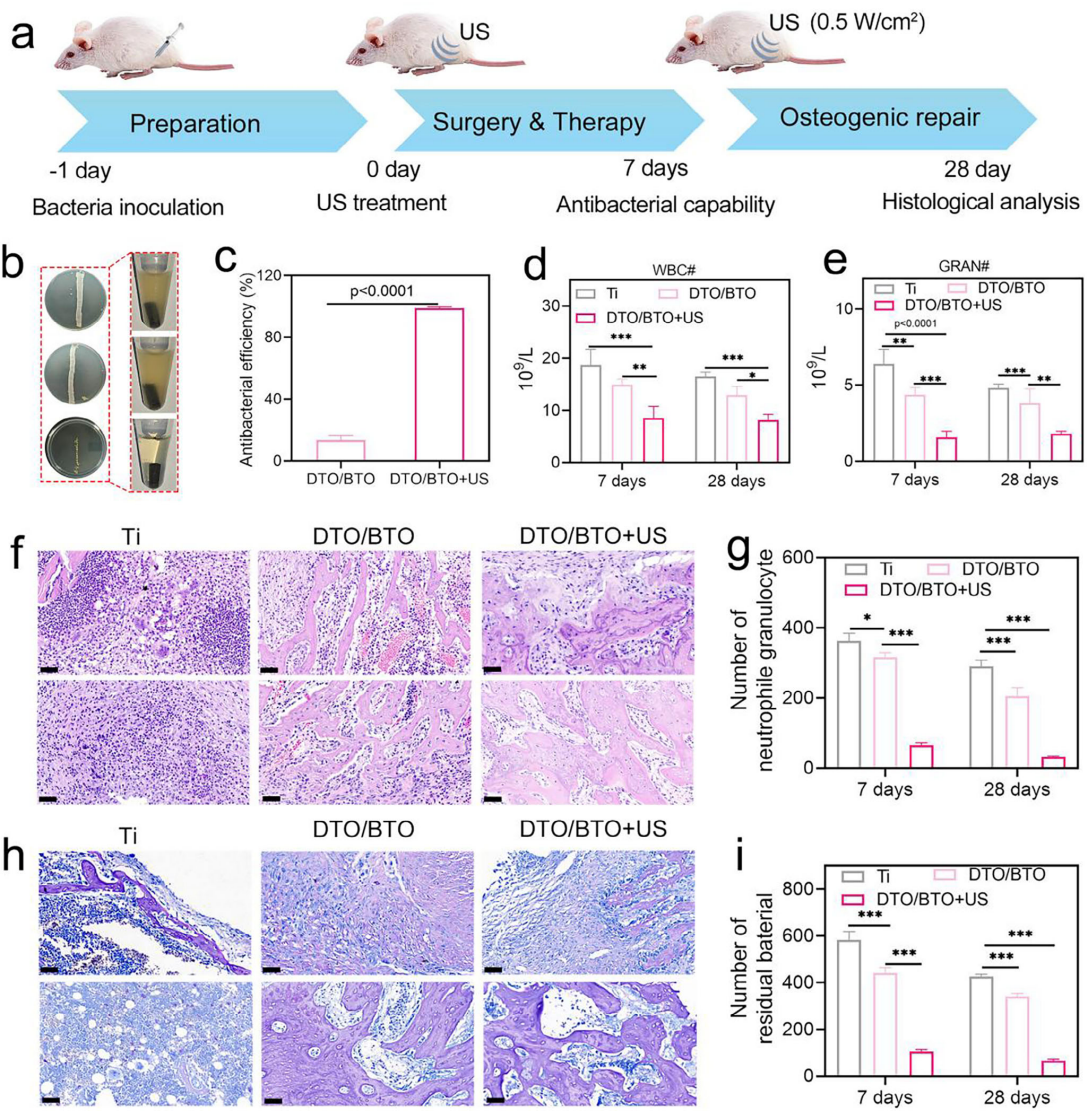
DTO/BTO for the treatment of *S. aureus* osteomyelitis. a) A diagram of the experimental design. b) in vivo antibacterial ability of bacteria‐infected samples after implantation for 7 days. c) the corresponding antibacterial efficiency of bacteria extracted from the infected implants and incubated for 24 h. d) WBC and (e) gran counts from blood from the different groups tested at 7 and 28 days postsurgery. f) H&E staining of different groups on 7 and 28 days, and g) corresponding H&E staining statistical chart (*n* = 3 independent samples per group, scale bar = 50 µm). h) Giemsa staining of different groups on 7 and 28 days, and i) corresponding S. aureus statistical chart (*n* = 3 independent samples per group, scale bar = 50 µm).(The error bars indicate means ± SD. *p < 0.05, **p < 0.01, ***p < 0.001).

The favorable antibacterial effect of DTO/BTO in vitro and its ability to promote cellular osteogenic differentiation led us to investigate its effects in an established bacterial‐infected bone defect model. Our goals were to evaluate the therapeutic effect of DTO/BTO on infected bone defects under ultrasound excitation and to assess the osseointegration ability of DTO/BTO. **Figure** **7**a shows a schematic diagram of the different treatments performed on SD rats during the 28‐day treatment period. Before the operation, the intervention was performed on the tibial part of the rats, a bone defect was modeled by drilling holes, then bacterial infection was carried out, and DTO/BTO implants were implanted into the defect site. After successful modeling, US treatment was performed on day 0. In order to detect the antibacterial effect, relevant tests, such as blood routine and H&E staining, were performed on day 7. In order to evaluate the osteogenic effect, histological analysis was performed on day 28 after US treatment. The different groups of materials were incubated for one day after implantation at the site of infection, and then the rats were treated with 20 min of US treatment at the site of the bone defect to assess the in vivo antimicrobial properties of the implants. As shown in Figure 7b,c, the implants were removed from the bone defect site, spread on a plate, incubated for 24 h, and then photographed. The removed implants were also placed in LB medium and incubated in an incubator at 37 °C for 24 h. The OD values were read at 600 nm using a multifunctional enzyme marker. The results showed that the number of bacterial colonies was much lower in the implants of DTO/BTO after US treatment than in the Ti control group. The calculated in vivo antimicrobial efficiency of DTO/BTO+US was 99.8% ± 0.20%. The 7‐day and 28‐day blood counts used to investigate the level of postoperative infections, as shown in Figure 7d,e, revealed high levels of leukocytes (WBC) and granulocytes (Gran) in both the Ti and DTO/BTO groups at 7 and 28 days, suggesting the presence of a strong inflammatory response induced by bacterial infection in the rats. By contrast, the DTO/BTO + US treatment group showed that the levels of these indicators were maintained in the normal range.^[^ ^6^, ^12^ ^]^

H&E and Giemsa staining were used to detect the inflammatory response in different groups after 7 and 28 days of treatment. The H&E staining sections of different groups (Figure 7f), and the corresponding statistics for neutrophil counts (Figure 7g) show that, after 7 days of treatment, the neutrophil counts were significantly lower in the DTO/BTO+US group than in the Ti and DTO/BTO groups, indicating that ultrasound treatment can reduce the inflammatory reaction at the infection site. The inflammatory reaction in the DTO/BTO+US group had basically disappeared after 28 days of treatment. The Giemsa‐stained sections of the bone defect sites are shown in Figure 7h, while Figure 7i shows the statistically obtained number of *S. aureus* present at the bone defect site after 7 and 28 days of treatment. The number of S. aureus was greater in the Ti group than in the DTO/BTO group. After 28 days of treatment, the bacteria in the DTO/BTO+US group had basically disappeared, confirming that ultrasound‐excited DTO/BTO could effectively kill bacteria and reduce the inflammatory response of the wound.^[^ ^22^, ^29^ ^]^

**Figure 8:**
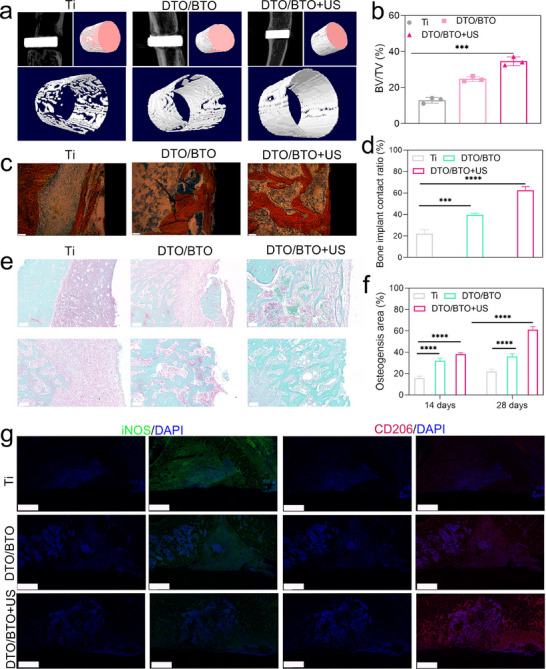
Bone integration of DTO/BTO in vivo. a) The 2D and 3D pictures of DTO/BTO and Ti6 groups measured by Micro‐CT. b) Bone volume (BV)/tissue volume (TV) values of different groups. c) Van Gieson's picrofuchsin staining (the scale bar is 200 µm) at 7 and 28 days, and d) bone implant contact of different samples. e) Safranin‐O and Fast Green staining (the scale bar is 100 µm), and f) osteogenesis area of different groups. (*n *= 3 independent experiments per group, *p < 0.05, **p < 0.01). g) immunofluorescence staining images of nucleus along with CD206 ang INOS expressed within the tissue surrounding the pillars implanted for 7 days. (*n* = 3, scale bar = 500 µm, *p < 0.05, **p < 0.01, ***p < 0.001).

**Figure** **8**a shows the microcomputed tomography (micro‐CT) results for bone regeneration in each group at 28 days. The results after 3D reconstruction showed a lower expanse of new bone area in the Ti and DTO groups than in the DTO/BTO+US group. Taken together with the results of the in vivo antimicrobial experiments, bacterial infection was confirmed to severely disrupt bone regeneration. As shown in Figure 8b, the analysis of bone volume ([object volume]/TV [tissue volume]) revealed a much higher bone volume fraction of 34.6% in the DTO+US group than in the Ti group (12.9%) or the DTO group (24.8%).^[^ ^6^, ^30^ ^]^

Van Gieson's picro‐fuchsin staining and Safranin‐O/Fast Green staining were used to assess the histopathology of the implants after 28 days (Figure 8c–f). With van Gieson's picro‐fuchsin staining, red staining represents newly generated mineralized bone tissue. As shown in Figure 8c, contact with the bone was better for the DTO/BTO+US implant than for the Ti and DTO/BTO implants. Quantitative results (Figure 8d) showed that contact with the bone was better for the DTO/BTO+US group (62.6%) than for the Ti (22.2%) and DTO/BTO groups (39.7%). As shown in Figure 8e,f, Safranin‐O/Fast Green staining, used to assess osteogenic (green) or chondrogenic differentiation (red), revealed a significantly larger osteogenic area around the implant at 7 and 28 days for the DTO/BTO+US group than for the other groups. Figure S22 (Supporting Information) presents the pull‐out test of the implants from the bone after 28 days of treatment. The analysis indicates that the ultrasound‐assisted implants exhibit excellent interfacial adaptation with the bone and demonstrate strong osseointegration ability.

The osteogenesis rate (obj./total) of the whole tissue was calculated as 38.3% and 61.2% for the 7‐day and 28‐day DTO+US groups, respectively. Immunofluorescence staining of the wound site (Figure 8g) revealed the lowest expression of the M1 macrophage marker inducible nitric oxide synthase (iNOS) in this group due to the elimination of bacterial infection on the DTO/BTO implants by US irradiation. The expression of the M2 macrophage marker CD206 was also higher in the DTO/BTO+US group than in the other groups, suggesting that the DTO/BTO+US group was closer to M2 polarization after 28 days of treatment.^[^ ^7^ ^]^ M2‐polarized macrophages are closely associated with anti‐inflammatory and tissue regeneration, which facilitates bone regeneration. Next, we evaluated the changes in the elastic modulus of DTO/BTO before and after implantation to further analyze its biological behavior (Figure S23, Supporting Information). The results, as shown below, indicate that this material possesses excellent mechanical properties and long‐term stability. Finally, in vivo imaging of major organs (Figure S24, Supporting Information). To better understand the dynamic polarization of macrophages during the early stages of treatment, flow cytometry was employed to analyze M1‐type and M2‐type macrophage populations at different time points, as shown in Figure S25 (Supporting Information). In the early phase, the presence of bacterial infection led to an increased proportion of M1‐type macrophages, which play a critical role in clearing pathogens. Following ultrasound treatment for 7 days, the bacterial load was significantly reduced, and a phenotypic switch from M1‐type to M2‐type macrophages was observed, indicating a transition toward a tissue‐repairing and anti‐inflammatory phenotype.

In addition, we also supplemented the application of implants in the rabbit bone defect experiment. Figures S26a,b (Supporting Information) present the blood count results at 7 and 28 days postsurgery, which were used to assess the infection levels in rabbits. The results showed that the white blood cell (WBC) and granulocyte (Gran) levels in the Ti and DTO/BTO groups remained elevated above normal levels at both 7 and 28 days postoperatively, indicating a strong inflammatory response caused by bacterial infection. In contrast, the WBC and Gran levels in the DTO/BTO+US treatment group remained within the normal range. To further evaluate the inflammatory response, H&E and Giemsa staining analyses were performed on the wound sites at 7 days postoperatively. H&E staining results (Figure S26c, Supporting Information) revealed that at 7 days post‐surgery, the number of neutrophils in the DTO/BTO+US group was significantly lower than in the Ti and DTO/BTO groups, confirming that ultrasound treatment effectively alleviated inflammation at the infection site. Moreover, Giemsa staining results (Figure S26d, Supporting Information) illustrated the bacterial distribution at the bone defect site, showing that the bacterial load in the Ti group was significantly higher than in the DTO/BTO group. Notably, in the DTO/BTO+US group, most bacteria were eradicated by day 7 post‐surgery. This further confirms that ultrasound‐activated DTO/BTO can effectively eliminate bacteria and significantly reduce inflammatory responses at the wound site.

The implantable DTO/BTO biomaterial demonstrated excellent osseointegration and ultrasound‐assisted anti‐infection capabilities in a rat bone defect model, significantly accelerating osteogenic differentiation and promoting new bone formation, thereby enhancing bone integration. After validating the antibacterial and osteogenic differentiation‐promoting abilities of DTO/BTO in vitro, we extended our research to a rabbit model to investigate the osseointegration performance of ultrasound‐assisted DTO/BTO implants. Micro‐CT 2D and 3D reconstruction analyses revealed that the formation of new bone tissue around the implant was relatively limited in the pure Ti rod group. In contrast, the DTO/BTO+US group exhibited significantly enhanced new bone formation under ultrasound assistance (Figure S27a, Supporting Information). This phenomenon can be attributed to the effective clearance of bacterial infections surrounding the implant and its environment under ultrasound stimulation, as well as the micro‐/nano‐structured surface of DTO/BTO promoting osteoblast proliferation. Quantitative analyses of bone parameters, including bone volume fraction (BV/TV), trabecular number (Tb.N), trabecular thickness (Tb.Th), and trabecular separation (Tb.Sp), further validated these findings (Figure S27b–e, Supporting Information). Specifically, the DTO/BTO+US group exhibited a higher BV/TV (Figure S27b, Supporting Information) value within the same volume of interest (VOI) and displayed optimal trabecular bone structure characteristics, suggesting that ultrasound‐assisted DTO/BTO is more conducive to peri‐implant bone regeneration than other biomaterials. In summary, after 28 days of ultrasound‐assisted DTO/BTO treatment, the infection at the rabbit wound site was effectively eradicated, and ultrasound significantly promoted new bone formation at the implant‐bone interface, ultimately achieving efficient osseointegration.

## 3 Conclusion

In summary, we successfully fabricated an ultrasound‐responsive implant coating using laser cladding technology; the coating enhanced piezoelectric catalysis for the treatment of bacterial infectious bone defects through spin polarization. The mechanism of ROS generation under ultrasound irradiation was elucidated using DFT calculations in conjunction with the experimental results. As a sonosensitizer, TiO_2_ can be excited by ultrasound (US) to produce sonoelectrons. The presence of oxygen vacancies reduces the bandgap, further facilitating electron generation. The abundance of Ti^3+^ species in DTO enhances spin polarization in DTO, such that the spin‐polarized electrons inhibit the recombination of electron‐hole pairs. The presence of a non‐centrosymmetric structure in BTO generates polarization under US excitation, forming an intrinsic electric field. Consequently, when DTO and BTO form a heterojunction, interfacial electrons accelerate the transfer of piezoelectric charges, thereby enhancing ROS production. In the present study, under US excitation, the antibacterial efficiency of DTO/BTO reached 99.83%, effectively inhibiting bacterial biofilm formation on the implant surface. DTO/BTO exhibited excellent biocompatibility, with no significant toxicity observed in vitro or vivo. Furthermore, the rough surface structure of DTO/BTO provided a conducive environment for cell growth. RNA sequencing indicated that the US‐induced current promoted Mitochondrial fusion by upregulating the expression of the MFN2 and OPA1 genes, thereby enhancing bone regeneration. In vivo experiments demonstrated that DTO/BTO exhibited excellent bone integration following US treatment. In conclusion, DTO/BTO combined with US irradiation shows significant potential for the effective treatment of deep tissue infections in bone defects.

## 4 Experimental Section

### Preparation of the DTO and DTO/BTO

Titanium (Ti) was purchased from Shunhang Metal Materials Co. (China). BaCO_3_ nanopowder was obtained from Shanghai Aladdin Biochemical Technology Co., Ltd (China). First, pure titanium was polished smooth with different grits of (240, 600, 800, and 1200) silicon carbide (SiC) sandpapers, after which it was ultrasonically cleaned and put into an ethanol solution for spare. Next, a BaCO_3_ solution with a concentration of 5 mg mL^−1^ and spin‐coated onto the treated titanium surface and dried in an oven at 60 °C for 2 h. Different samples were coated using a laser generator (JHM‐1GY‐300B; Lumonics). With a wavelength of 1.06 µm, laser cladding was carried out by a CW 2 kW Nd: YAG laser, with optimal parameters of laser current = 115 A, pulse width = 2 ms, frequency = 20 Hz, spot diameter = 0.6 mm, and scanning speed = 5 mm s^−1^. DTO is synthesized in the same way as DTO/BTO, except that the titanium surface is not spin‐coated with BaCO_3_.

### Surface Characterization

The microscopic morphology of the obtained samples was detected by scanning electron microscopy (FE‐SEM JSM7100F, JEOL, JP). TEM images were obtained using a Tecnai G20 (FEI, Co., USA). XRD (D8A25, Bruker, Germany) was used to detect the crystalline phase of the samples. The elemental composition of the samples was tested by X‐ray photoelectron spectroscopy (XPS) (ESCALAB 250Xi, Thermo Scientific, USA) spectra. The UV–vis absorption spectra were measured by UV−vis−NIR spectrometer (UV‐3600, Shimadzu, Japan). Ultrasound treatments were conducted using an Intelect Mobile Ultrasound (Chattanooga 2776, DJO Group, USA). The water contact angles of the samples were measured at room temperature using the JC2000D Contact Angle System (POWEREACH, China). Piezoresponse force microscopy (PFM) measurements were conducted using an atomic force microscope (AFM, Bruker Multimode 8) equipped with a ferroelectric test system.

### Ultrasonic Electrochemistry Measurement

The ultrasonic electrochemistry measurements for all samples were conducted using a three‐electrode CHI 660E electrochemical workstation. Platinum served as the counter electrode, and an Ag/AgCl electrode was used as the reference electrode. Ultrasound irradiation at 1.5 W cm^−2^ was employed as the excitation source, with a 0.5 m Na_2_SO_4_ solution used as the electrolyte.

### Sonodynamic and ROS Detection

To detect ROS production under ultrasound irradiation, a ROS Assay Kit utilizing 2′,7′‐dichlorofluorescein (DCFH) was employed. This compound reacts with ROS to produce fluorescence, which can be measured using a microplate reader. ROS signals were also detected using electron spin resonance (ESR). For the DTO/BTO samples treated with ultrasound for 10 min, the signal of ^1^O_2_ was trapped using TEMP and detected via ESR. Similarly, for the DTO samples treated with ultrasound for 10 min, 5,5‐dimethyl‐1‐pyrroline N‐oxide (DMPO) trapped the signal of ⋅OH, which was then detected by ESR.

### Theoretical Calculations

First‐principles calculations based on density functional theory (DFT) were utilized within the generalized gradient approximation (GGA) framework, employing the Perdew‐Burke‐Ernzerhof (PBE) functional. The projected augmented wave (PAW) method was applied to represent ionic cores, while valence electrons were treated using a plane‐wave basis set with a 400 eV kinetic energy cutoff. Gaussian smearing with a width of 0.05 eV was used to allow partial occupancies of the Kohn−Sham orbitals. Electronic self‐consistency was achieved when energy variations dropped below 10^−5^ eV, while geometry optimization was deemed converged when the energy change was under 0.05 eV Å^−1^. A vacuum spacing of 15 Å was maintained perpendicular to the structural plane. Brillouin zone sampling employed a 2×2×1 Monkhorst‐Pack k‐point mesh. Adsorption energies (Eads) were determined using E_ads_ = E_ad/sub_ – E_ad_ – E_sub_, where Ead/sub, Ead, and Esub correspond to the total energies of the adsorbate‐substrate system, isolated adsorbate, and clean substrate, respectively. The free energy was evaluated as G = E + ZPE – TS, incorporating total energy from DFT calculations, zero‐point energy, and entropic contributions.

### In Vitro Antibacterial Assay

To evaluate the in vitro antibacterial performance of DTO/BTO, the Staphylococcus aureus strain (ATCC 29 213) was used as the test organism. A spread plate method was employed to observe the antibacterial effect. The samples were first sterilized under UV irradiation for 30 min. Next, the bacterial suspension was diluted to 10^7^ CFU mL^−1^ in a test tube (4 mL) and then transferred onto the surface of the samples. The samples were then exposed to ultrasound irradiation for 20 min. After treatment, the *S. aureus* samples were further diluted, and 20 µL of each was spread on Luria‐Bertani (LB) agar plates and incubated at 37 °C for 24 h. Each sample was prepared in triplicate. The antibacterial performance of Ti, DTO, and DTO/BTO was assessed using this method. The number of bacterial colonies (N) on the plates was counted, and the antibacterial rate was calculated.The formula is as follows: Antibacterial efficiency (%) = (*N*_Control group_‐*N*_Experimental group_)/*N*_Control group_ *100%

To observe the morphology of the bacteria after the antibacterial assay, a field emission scanning electron microscope (FE‐SEM) was used. The samples and bacteria were treated in the same manner as in the spread plate test. After ultrasound treatment, the samples were allowed to stand for a period of time, the supernatant was removed, and the samples were washed three times with PBS. To immobilize the bacteria, 200 µL of 20% glutaraldehyde was added. After 2 h of fixation, the solution was removed, and the samples were washed three times with PBS. Finally, the samples were dehydrated sequentially with gradient alcohol (30%, 50%, 70%, 90%, and 100%) for 20 min each.

### Live/Dead Staining

Live/dead staining was performed according to the manufacturer's instructions. Briefly, bacteria were cultured with different samples and treated with ultrasound for 20 min. Then, the culture solution was replaced with EthD‐III working solution (Yeasen) and incubated for 20 min in the dark.

### Anti‐Biofilm Tests

Crystal violet was used to detect biofilms on samples. The samples were placed in 24‐well plates, and each well was inoculated with 500 µL of *S. aureus* suspension at a concentration of 10^9^ CFU mL^−1^. After 48 h of co‐culturing in an incubator at 37 °C, the samples were exposed to ultrasound for 20 min and then immersed for 2 h. The supernatant was discarded, and the sample surface was washed with PBS. Next, 200 µL of 0.1% (v/v) crystal violet solution was added, and the samples were stained for 20 min at 37 °C. After staining, the excess dye was washed away with PBS, and 1 mL of anhydrous ethanol was added to decolorize the samples for 2 h. Finally, the absorbance at 600 nm (OD600) was measured.

### Research Experiment on Antibacterial Mechanism

*S. aureus* Bacteria DNA Extract. A bacterial DNA kit was used to detect DNA damage in the different samples (Ti, DTO, DTO/BTO). Specifically, the bacteria were washed three times with deionized water (DI), and then the bacterial solution was centrifuged and diluted to 10^9^ CFU mL^−1^. Subsequently, 200 µL of the bacterial suspension was added to the surface of the samples and treated with ultrasound for 20 min. Finally, the bacterial DNA was extracted using the bacterial DNA kit according to the manufacturer's instructions.

### ATP Test

The Ti, DTO, and DTO/BTO samples were placed into separate wells of a 96‐well plate, and PBS‐cleaned *S. aureus* (at 10^7^ CFU mL^−1^) was added to each well. This was followed by ultrasound irradiation for 20 min. Bacteria from the different samples were then collected in PBS, lysed, and centrifuged. Finally, the fluorescence intensity in the supernatant was detected using an ATP kit (cat# S007, Beyotime, China).

### In Vitro Toxicity of the DTO/BTO

The cells were trypsinized using trypsin solution (Boster), and 10 000 cells per well were seeded into 24‐well plates. The diluted cells were added to wells containing Ti, DTO, and DTO/BTO samples and cultured in an atmosphere with 5% CO_2_ at 37 °C for 1 day. At 1, 3, and 7 days, the culture medium in each well was removed and replaced with a mixture of 10% CCK‐8 solution (Boster) and culture medium, according to the manufacturer's instructions. After 1 h of incubation at 37 °C, 100 µL of the liquid from each well was transferred into 96‐well plates to measure absorbance values using a microplate reader at room temperature.

The growth morphology of cells on Ti, DTO, DTO/BTO was observed by SEM. After 1, 3, and 7 days of co‐culture of cells and different samples, the cell samples were prepared by fixation and dehydration, and the treatment method was consistent with bacterial SEM. The cytoskeletons were incubated with FITC for 30 min, and the cytoskeletons were incubated with 4′,6‐diamidino‐2‐phenylindole (DAPI) staining solution according to the manufacturer's instructions, and then observed under a fluorescence microscope.

### Alizarin Red *S* Staining

Sterile samples were co‐cultured with MSCs cells in osteogenic induction solution. After 21 days, the medium was discarded, and the cells were washed three times with PBS and fixed with 4% paraformaldehyde for 30 min. Subsequently, the cells were stained with 0.2% Alizarin Red S solution (Solarbio) for 1 h at room temperature and then photographed.

### Quantitative Reverse Transcription Polymerase Chain Reaction (qRT‐PCR)

After co‐culturing the cells with completely sterile material, the cells were collected, and the RNA of MSCs was extracted using Trizol (Invitrogen, Carlsbad, CA, USA) reagent. PrimeScript RT Master Mix (TaKaRa, Japan) was used to synthesize cDNA from the extracted RNA. The samples were then subjected to RT‐qPCR using SYBR Premix EX Taq (TaKaRa, Japan). Osteogenesis‐related genes, including RUNX2, COL‐1, and OPN, were amplified and detected. Primer sequences for GAPDH, COL‐1, RUNX2, and OPN are shown in Supplementary Table S1.

### Rat Osteomyelitis Model in Vivo

The animal experiment was conducted in accordance with the Guide for the Care and Use of Laboratory Animals of the National Institutes of Health. Male Sprague Dawley rats (300–350 g body weight) were purchased from Tianjin Yishengyuan Biotechnology Co., Ltd. The animal experiments were approved by Tianjin Yishengyuan Biotechnology Co., Ltd, and were performed in strict compliance with the regulations proposed by the Ministry of Health of the People's Republic of China (YSY‐DWLL‐2023368).

After the rats were in good growth conditions and had no adverse reactions, they were divided into three groups: Ti group, DTO/BTO group, and DTO/BTO+US, and then treated with osteomyelitis modeling. First, all instruments and samples were sterilized before the surgery. Then, after anesthetizing the rats with 3% sodium pentobarbital, the rats were dehairing and sterilizing the lower part of the right knee, opening up the muscle tissue, making a hole of 1.5 mm in diameter in the exposed area of the bone with an electric drill, and then performing the bacterial modeling (10^9^ CFU), implanting the samples, followed by suturing the wounds. In the DTO + US group, ultrasound treatment was performed for 20 min. During the treatment, the temperature was detected with a thermal image, and the temperature was not more than 45 °C. All animals were raised in the same environment and fed in the same way. After the surgery, the experimental animals were euthanized. In order to observe if a biofilm forms on the surface of the implant, they were removed after 2 weeks of treatment and coated with plates. The removed samples were incubated in LB medium for 24 h, and the OD value of the medium was read using an enzyme marker. After 7 days, the tissues from the infected sites were removed for embedding, and the tissue sections were processed, after which the bacterial infections in the bone tissue sites were detected by H&E, GIEMSA staining. Routine blood examination involved collecting peripheral blood from the orbital venous system of rats in different groups using heparinized capillary blood‐collecting vessels (Hirschmann). The number of white blood cells (WBCs) and neutrophil granulocytes was evaluated using an animal hematology analyzer (BC‐2800vet).

### Bone Micro‐CT and Histopathological Evaluation

Quantification of gross bone morphology and microarchitecture was conducted using a BRUKER Micro‐CT SkyScan 1176 imaging system. 3D reconstruction images were generated and analyzed with SkyScan CTAnalyser software. Simultaneously, Safranin‐O/Fast Green staining was used to evaluate the implant surface for osteogenesis and chondrogenesis. Green areas indicated osteogenic regions, while red areas indicated chondrogenic regions. The percentage of osteogenesis within 20 µm from the implant surface was used to define the osteogenic rate. Van Gieson's picrofuchsin staining was employed to analyze mineralized bone tissue (red) around the implant/bone interface.

### Statistical Analysis

The P‐values were determined by unpaired t‐test, one‐way analysis of variance (ANOVA) with Dunnett's multiple comparisons test, or two‐way ANOVA with Dunnett's multiple comparisons test or with Sidak's multiple comparisons test. And the data were presented as mean values + standard deviations (SD). The value of SD indicates the error bar. When *p *< 0.05, it was considered statistically significant. The *n* number of biologically independent samples in each group was at least three. Exact n values are marked in the images.

## Conflict of Interest

The authors declare no conflict of interest.

## Supporting information



Supporting Information

## Data Availability

The data that support the findings of this study are available from the corresponding author upon reasonable request.
