# Supplementary material for: Accelerating Sono‐Piezoelectric Charge Transport for Antibacterial Therapy and Bone Regeneration by Metal‐Deficient TiO2 Spin‐Polarization Effect
Source: Adv Sci (Weinh). 2025 May 28;12(30):e03186. doi: 10.1002/advs.202503186 (PMC12376564; doi:10.1002/advs.202503186)
Supplement: Supplementary file 1 — Supporting Information [file ADVS-12-e03186-s001.docx]

**Supporting information**

**Accelerating sono-piezoelectric charge transport for antibacterial therapy and bone regeneration by metal-deficient TiO_2_ spin-polarization effect**

Chaofeng Wang, Shuilin Wu^*^, Congyang Mao, Liguo Jin, Hanpeng Liu, Yufeng Zheng, Chunyong Liang*, Shengli Zhu, Zhaoyang Li, Hui Jiang, Xiangmei Liu^*^.

Mr. C. Wang, Prof. X. Liu and Prof. C. Liang,

School of Health Science and Biomedical Engineering, Hebei University of Technology, Tianjin 300131, China

E-mail: [liuxiangmei@hebut.edu.cn](mailto:liuxiangmei@hebut.edu.cn) (X. Liu); [liangchunyong@hebut.edu.cn](mailto:liangchunyong@hebut.edu.cn) (L. Liang)

Prof. S. Wu, Prof. Y. Zheng

School of Materials Science & Engineering, Peking University, Yiheyuan Road 5#, Beijing, 100871, China

E-mail: slwu@pku.edu.cn (S. Wu)

Prof. C. Mao, Prof. S. Wu

Biomedical Materials Engineering Research Center, Hubei Key Laboratory of Polymer Materials, Ministry-of-Education Key Laboratory for the Green Preparation and Application of Functional Materials, School of Materials Science & Engineering, State Key Laboratory of Biocatalysis and Enzyme Engineering, Hubei University, Wuhan 430062, China

E-mail: slwu@pku.edu.cn (S. Wu)

Miss. Jin, Mr. H. Liu, Prof. S. Wu, Prof. S. Zhu, Prof. Z. Li, Prof. H. Jiang

School of Materials Science & Engineering, the Key Laboratory of Advanced Ceramics and Machining Technology by the Ministry of Education of China, Tianjin University, Yaguan Road 135#, Tianjin, 300072, China

E-mail: slwu@pku.edu.cn (S. Wu)

*Corresponding author: [slwu@pku.edu.cn](mailto:slwu@pku.edu.cn) (S. Wu); [liuxiangmei1978@163.com](mailto:liuxiangmei1978@163.com) (X. Liu); [liangchunyong@hebut.edu.cn](mailto:liangchunyong@hebut.edu.cn)


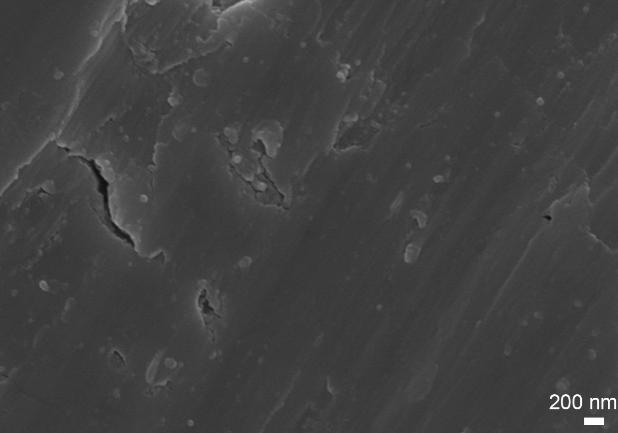

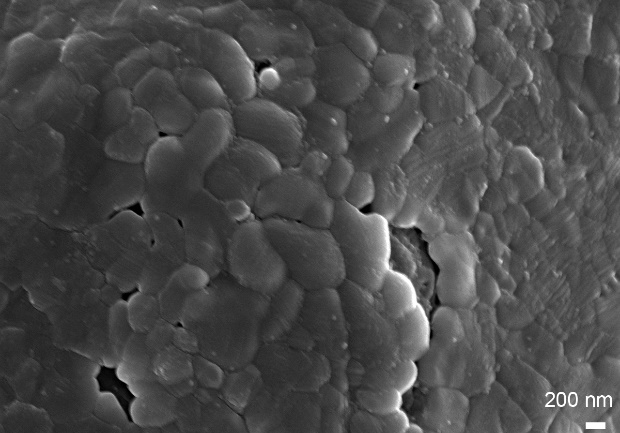

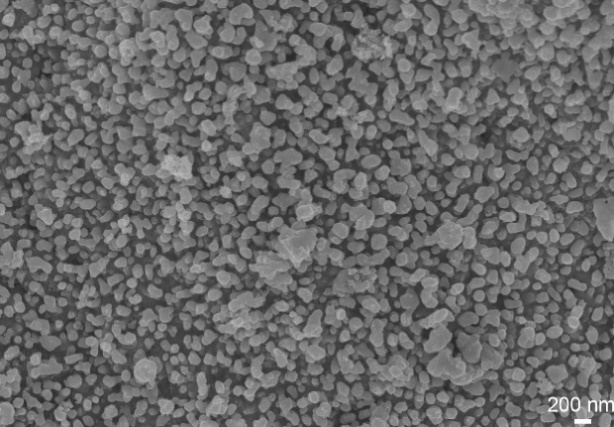


b

a

c

Figure S1. Scanning electron microscope (SEM) of (a) Ti, (b) DTO, (c) DTO/BTO.


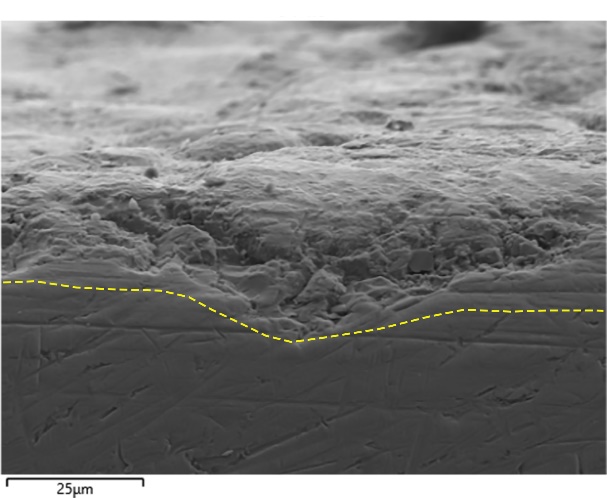

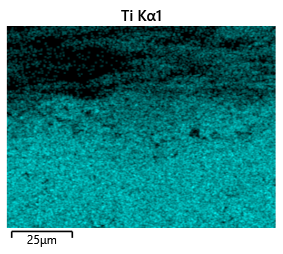


d

c

b

a


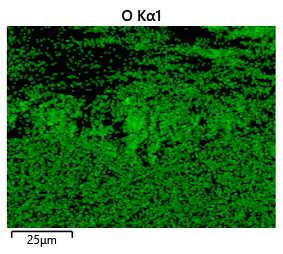

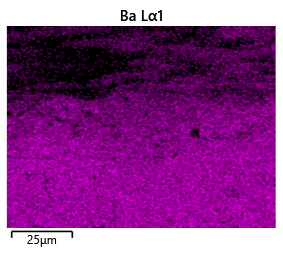


Figure S2. a) Cross-section SEM image of the DTO/BTO, the element mapping of the b) Ti, c) O, d) Ba of Cross-section SEM.


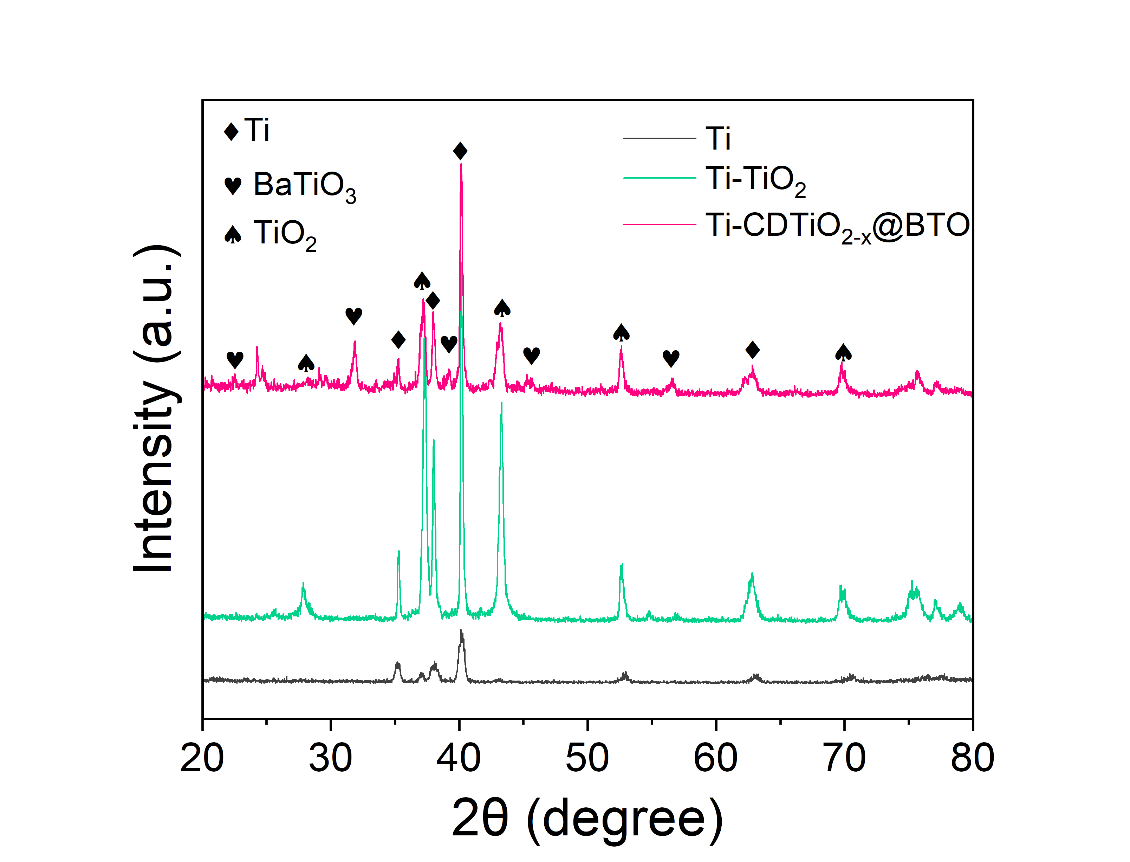


Figure S3. XRD patterns of Ti, DTO, DTO/BTO.


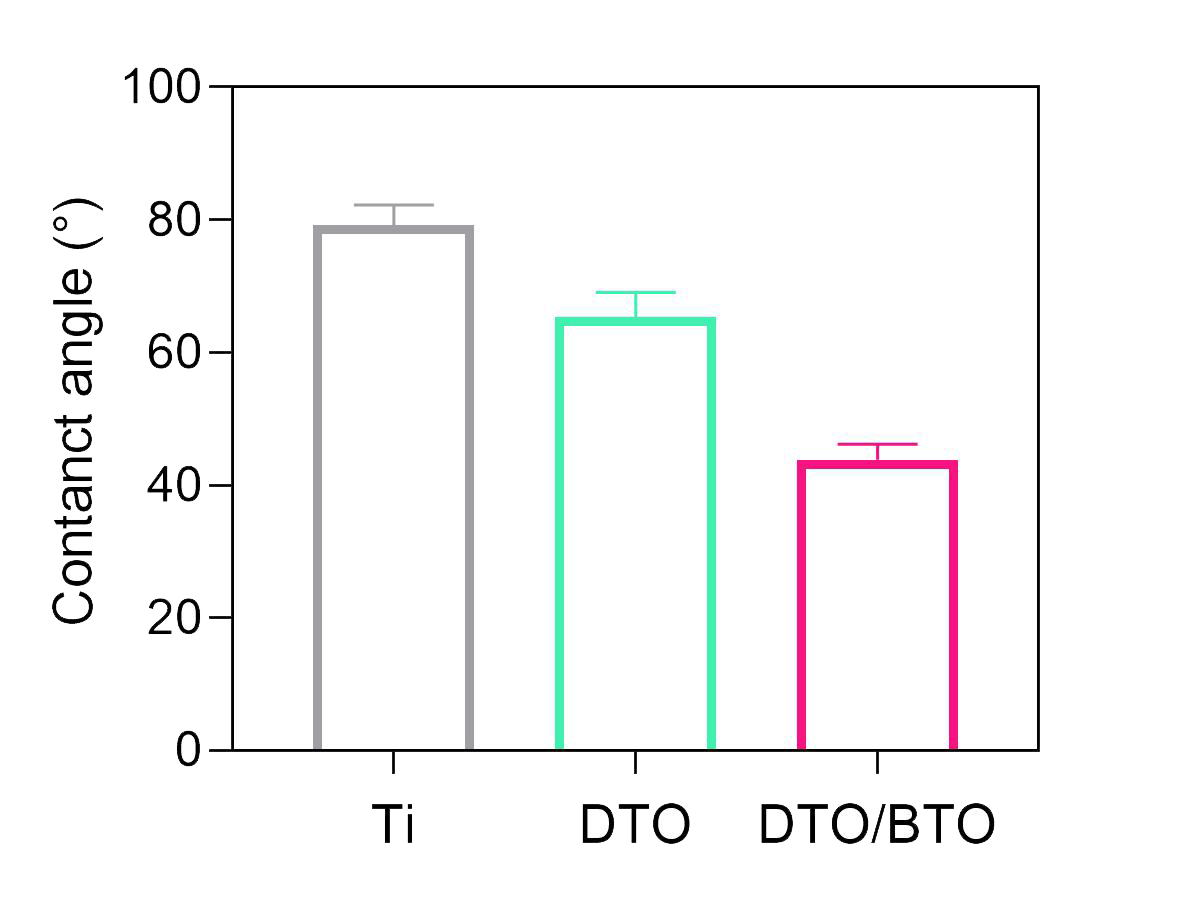


Figure S4. Contact angles of Ti, DTO, DTO/BTO.


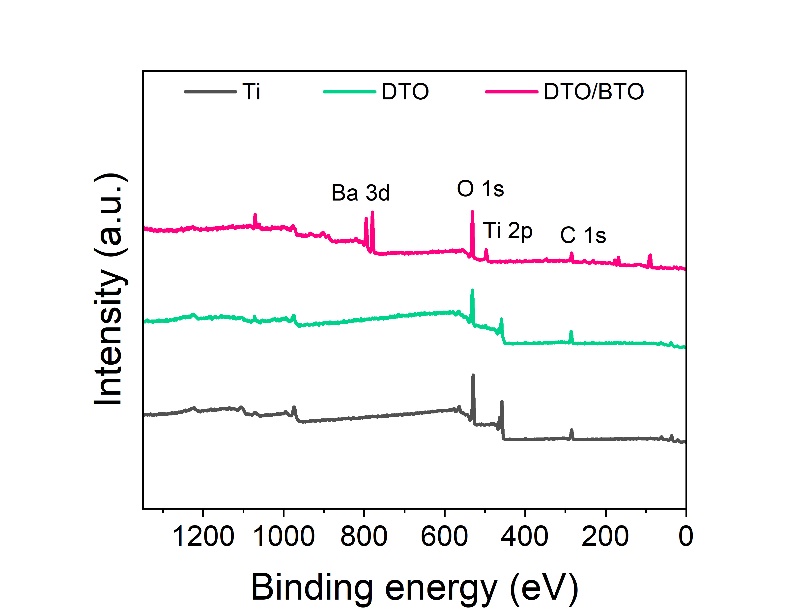


Figure S5. The survery spectra of Ti, DTO, DTO/BTO.


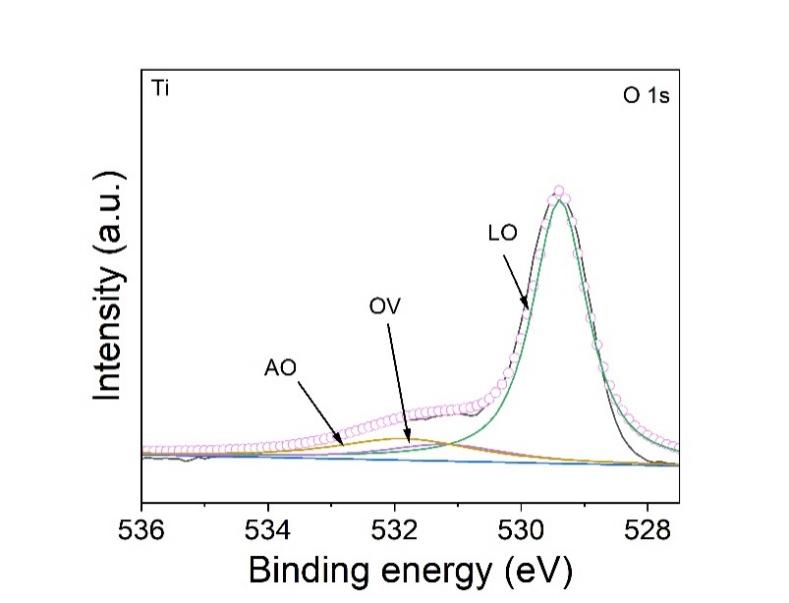

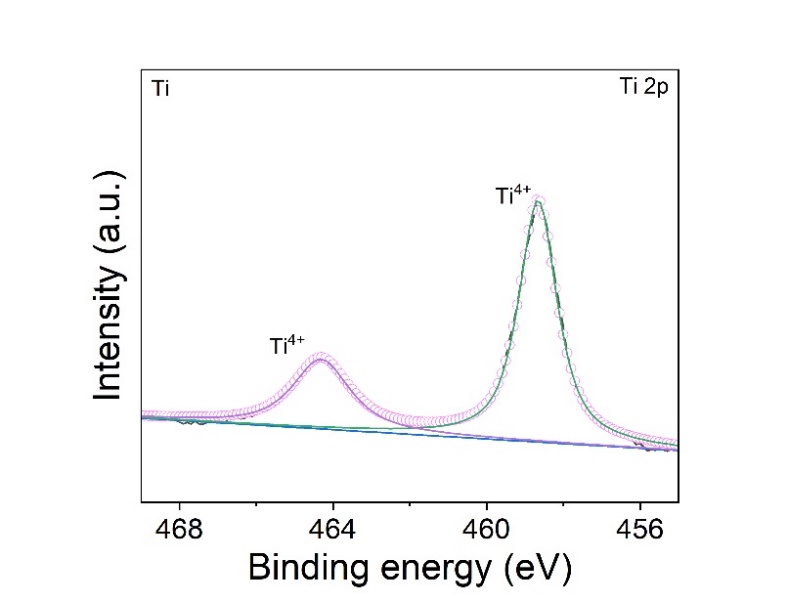


Figure S6. High-resolution XPS spectra of O1s and Ti 2p of Ti.


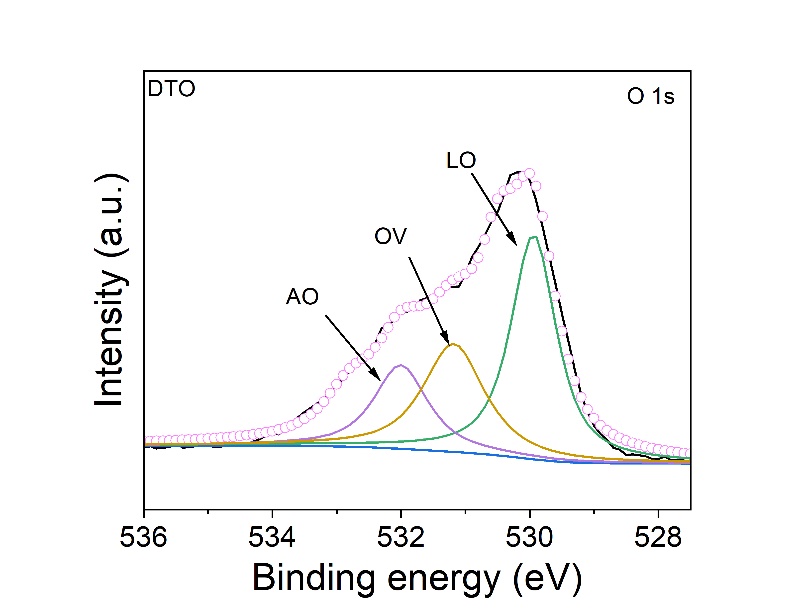

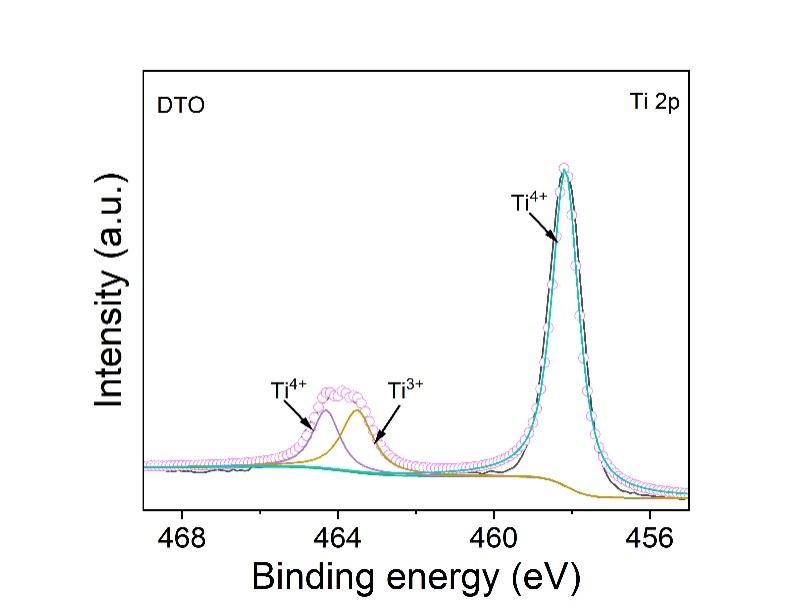


Figure S7. High-resolution XPS spectra of O1s and Ti 2p of DTO.


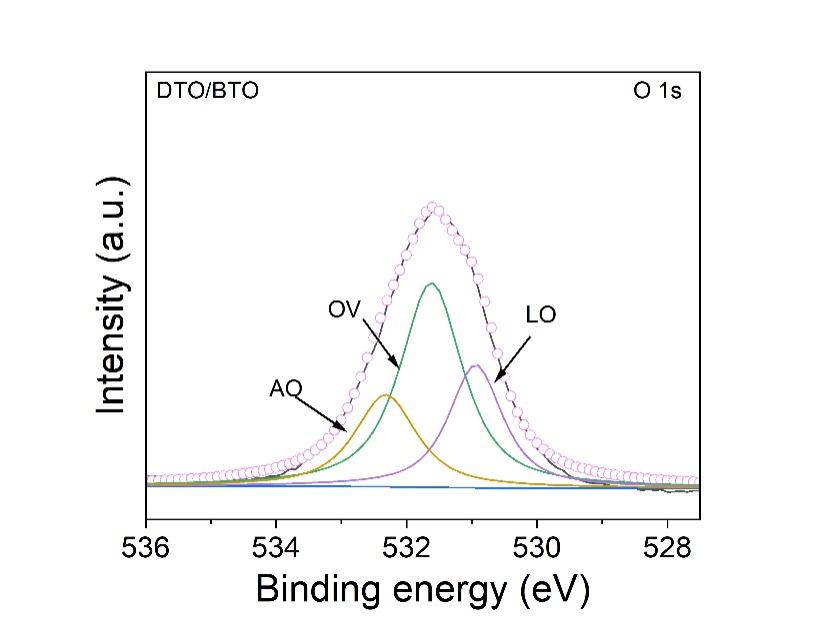

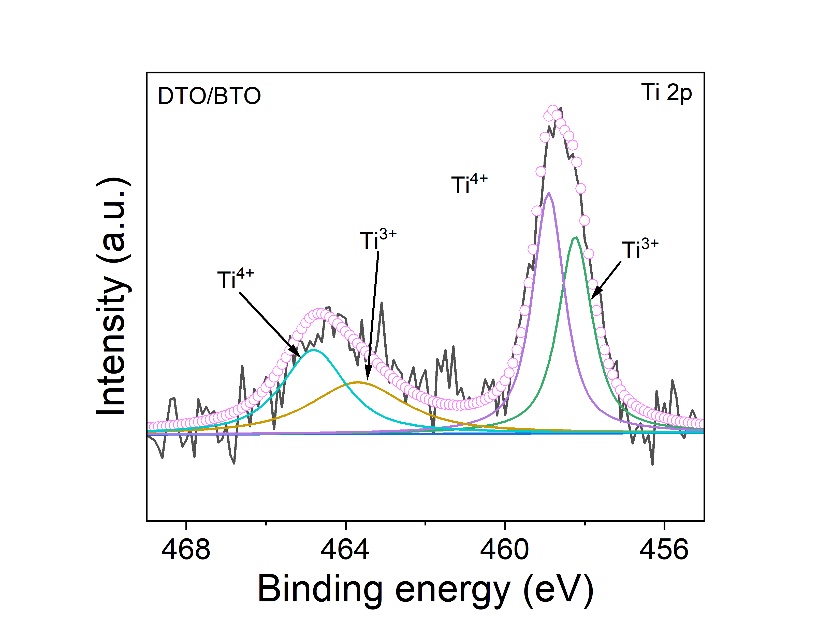

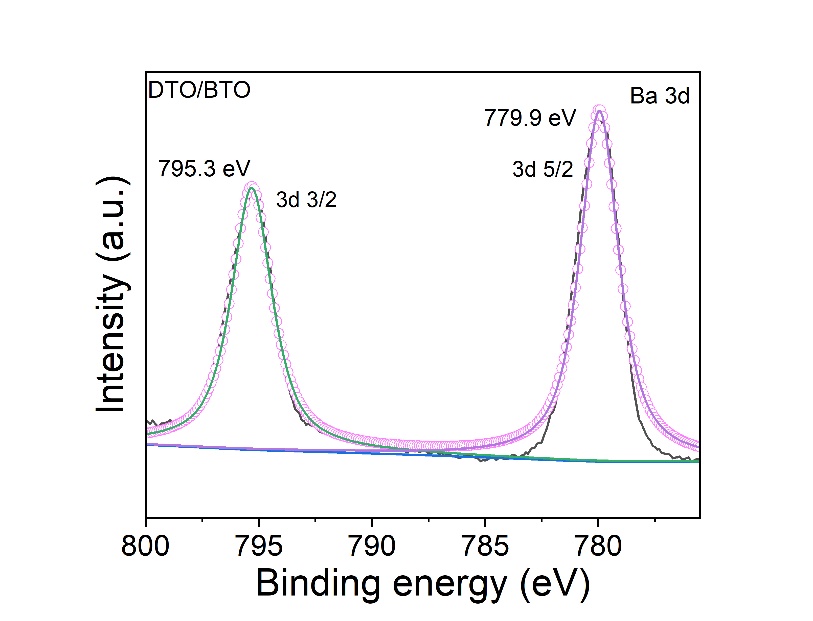


Figure S8. High-resolution XPS spectra of O1s, Ti 2p and Ba 3d of DTO/BTO.


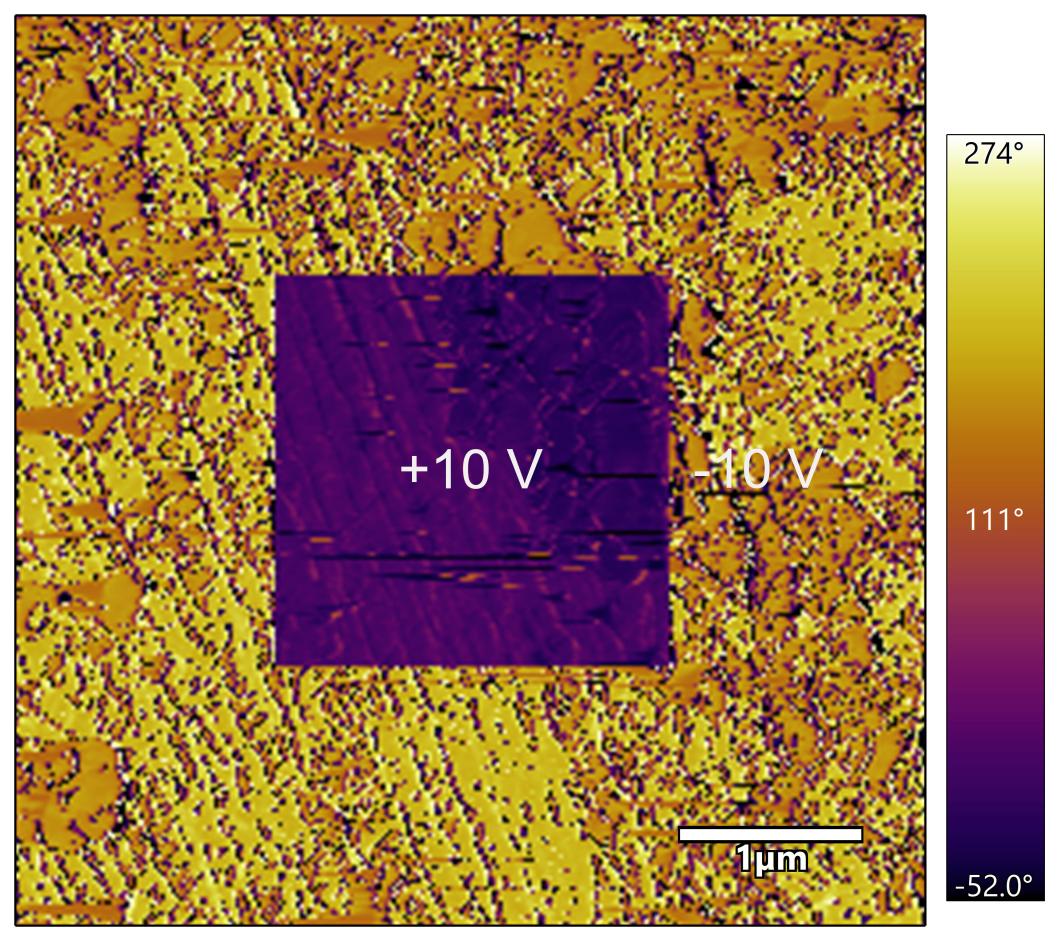


Figure S9. Local polarization inversion of DTO/BTO.


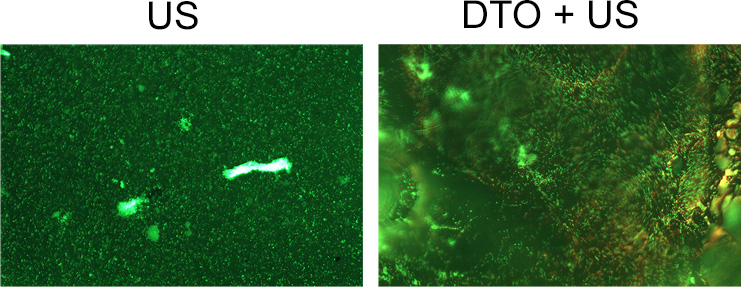


a


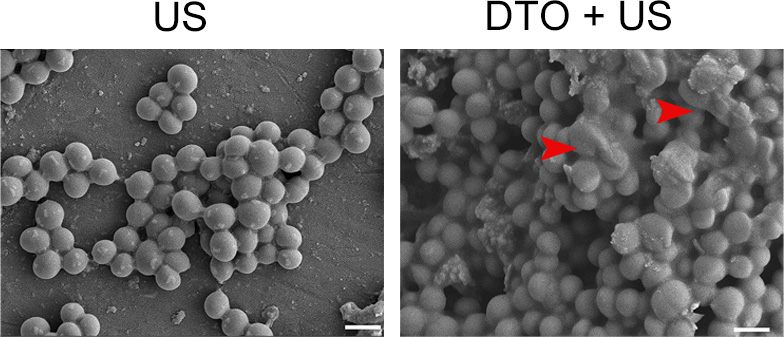


b

Figure S10 . (a) Fluorescent images of living (green) and dead (red) staining bacterial after treatment by US and DTO + US. (b) the SEM images of morphologies of *S. aureus* on different samples of US and DTO+US.


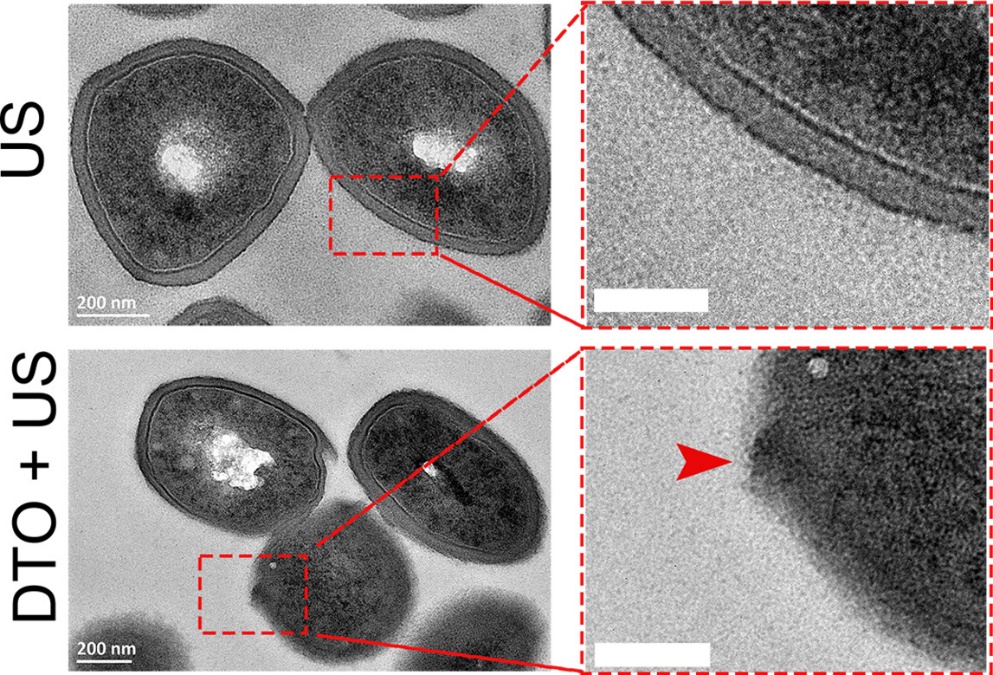


Figure S11. the TEM images of *S. aureus* treatments with US and DTO + US.


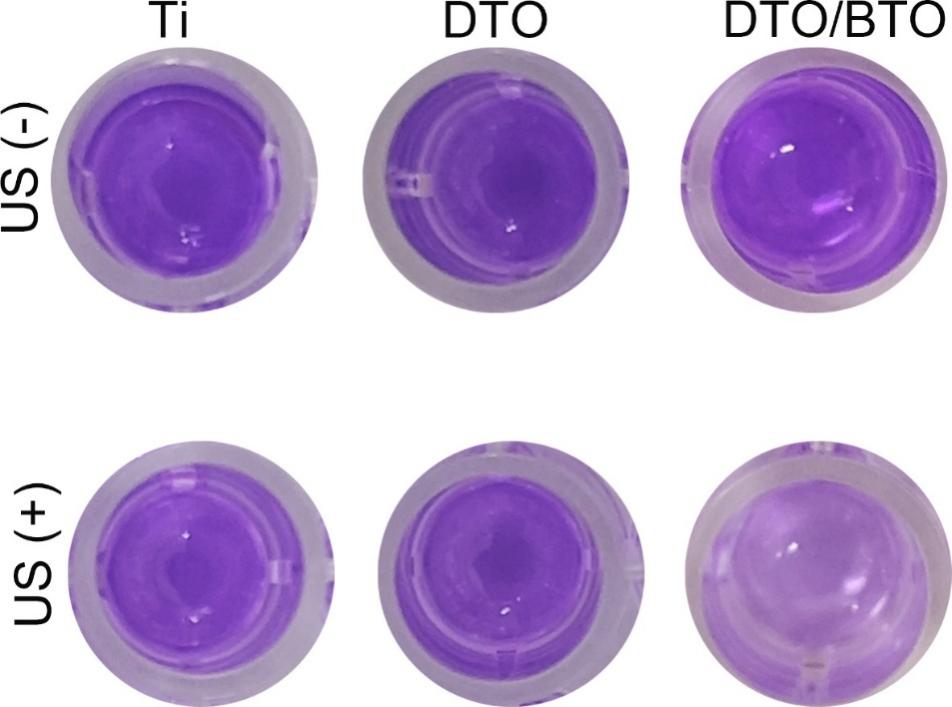


Figure S12. Experimental images of different groups of crystal violet.


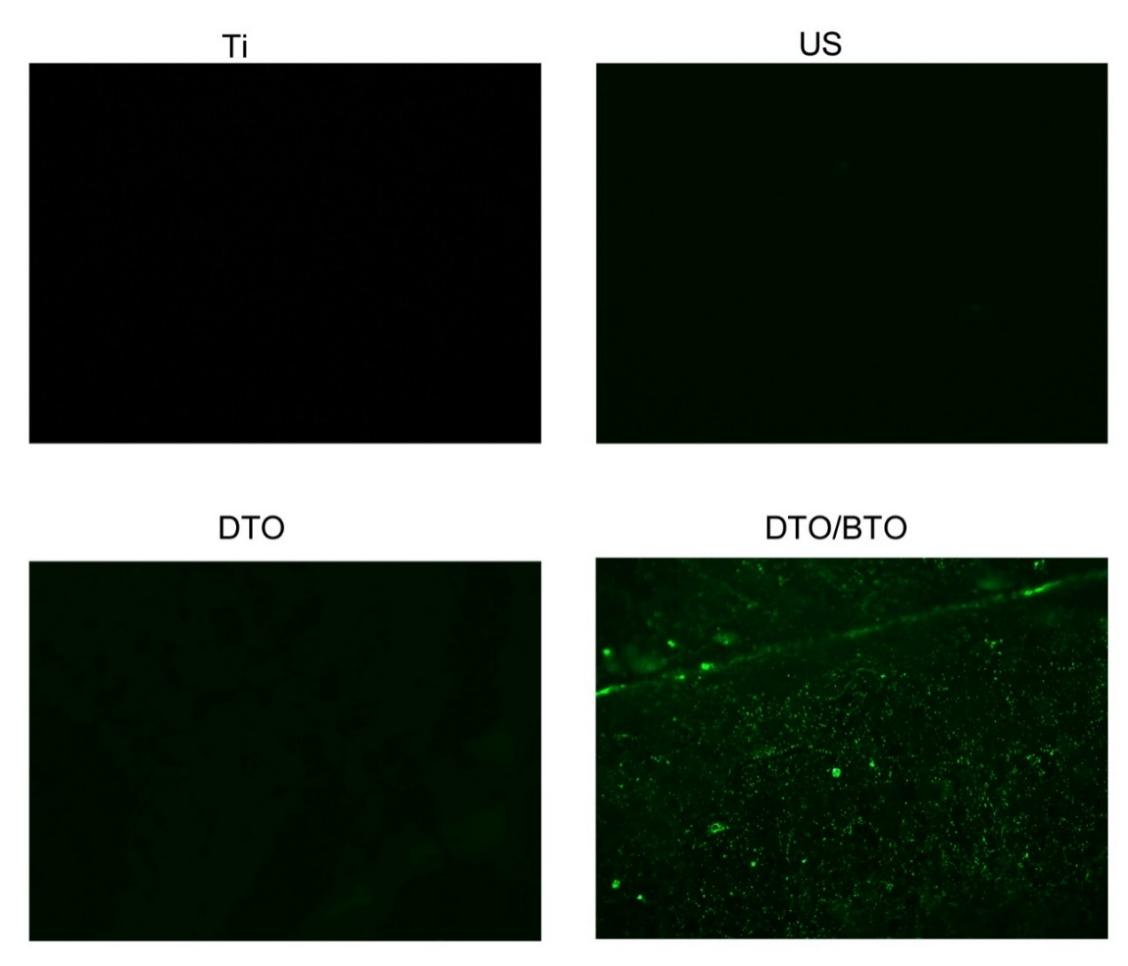


Figure S13. The fluorescence of S. aureus after treatments with a) Ti, b) US, c) DTO +US, d) DTO/BTO +US characterized by DCFH-DA probe.


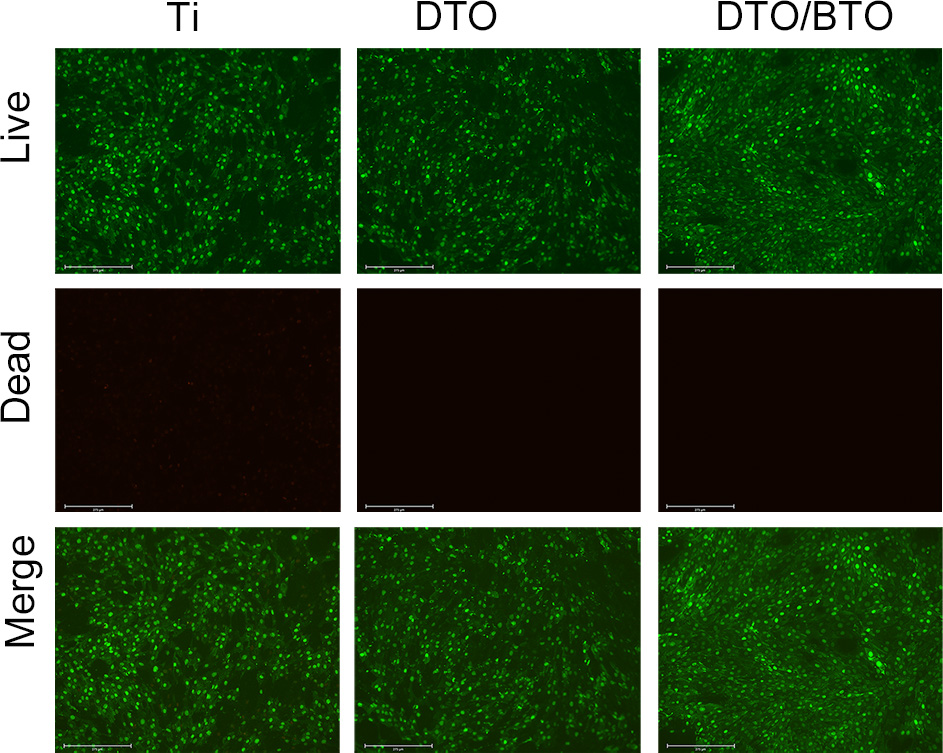


Figure S14. The Live/Dead staining assay treatment by Ti, DTO, DTO/BTO for 1 days.


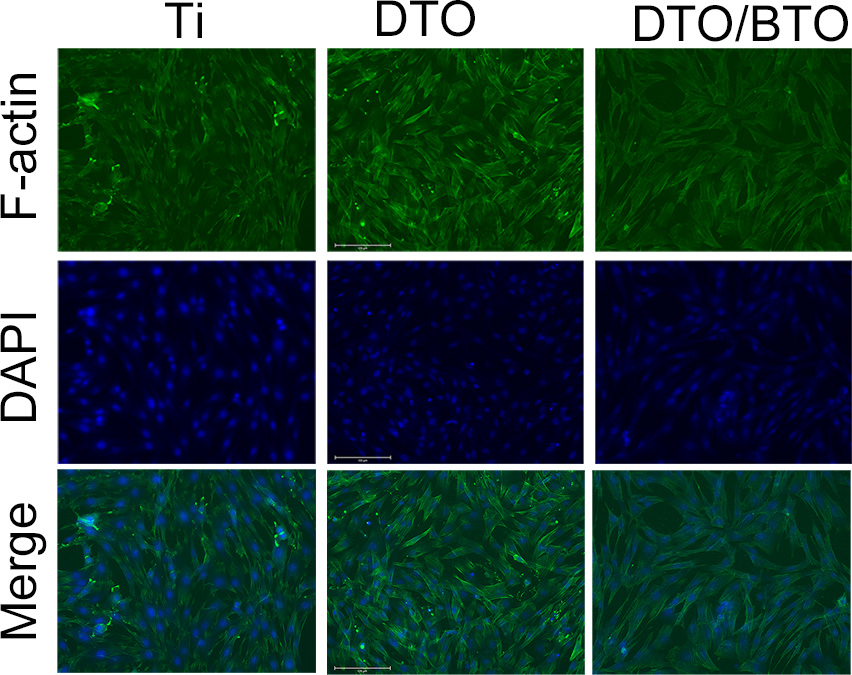


Figure S15. Fluorescence images of MSCs cocultured with different samples for 3 days.


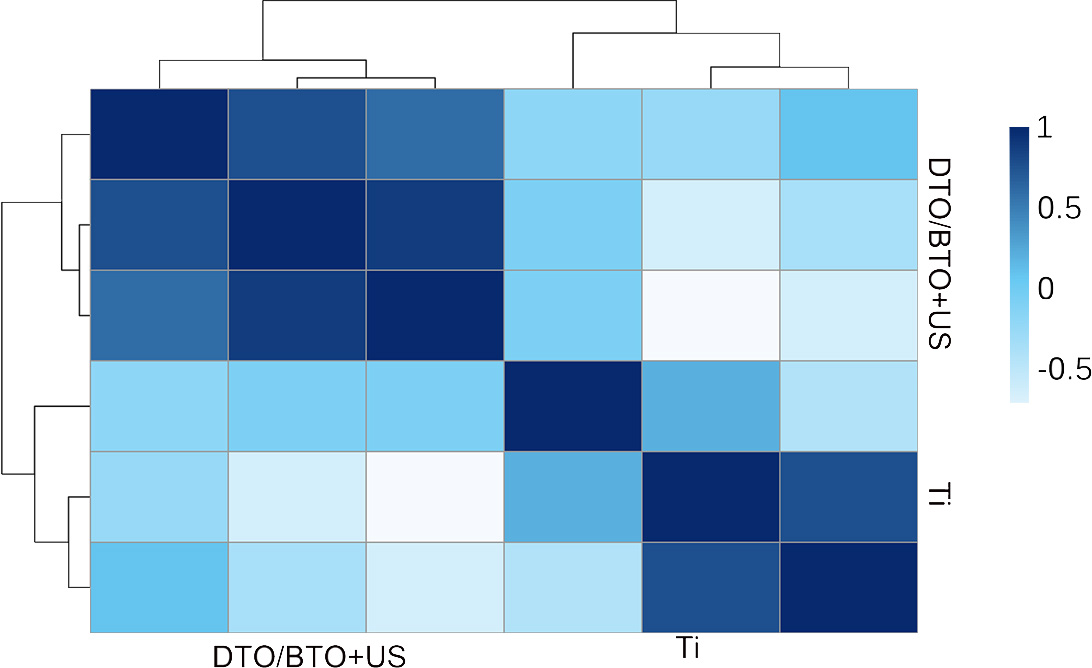


Figure S16. Heatmap of sample-to-sample distances.


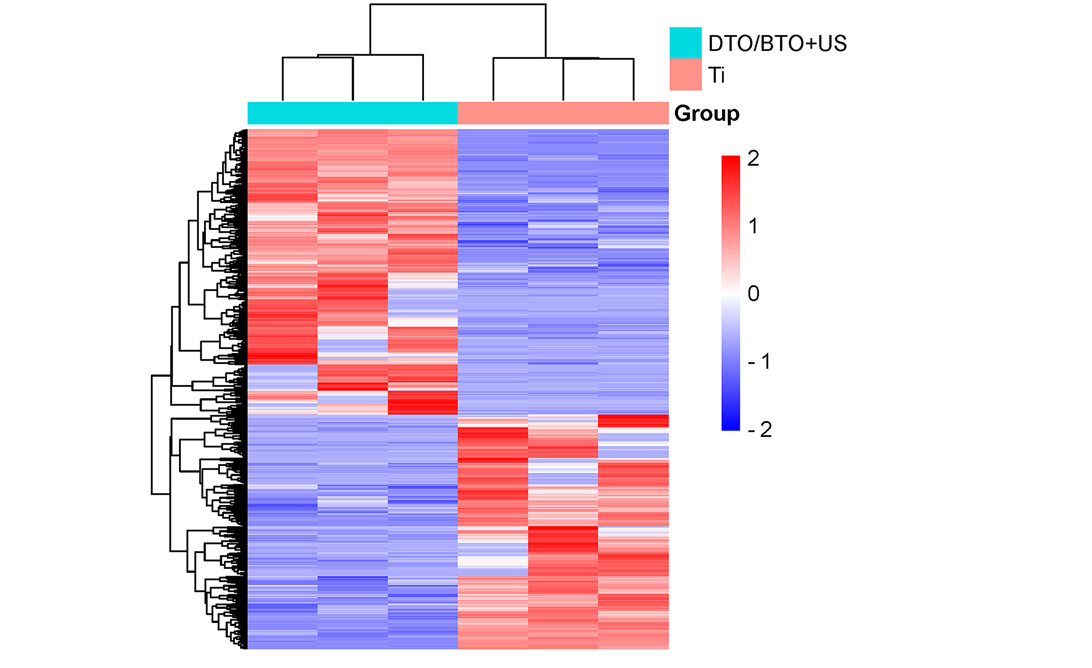


Figure S17. Heatmap of the diﬀerential genes between the Ti and DTO/BTO + US groups.


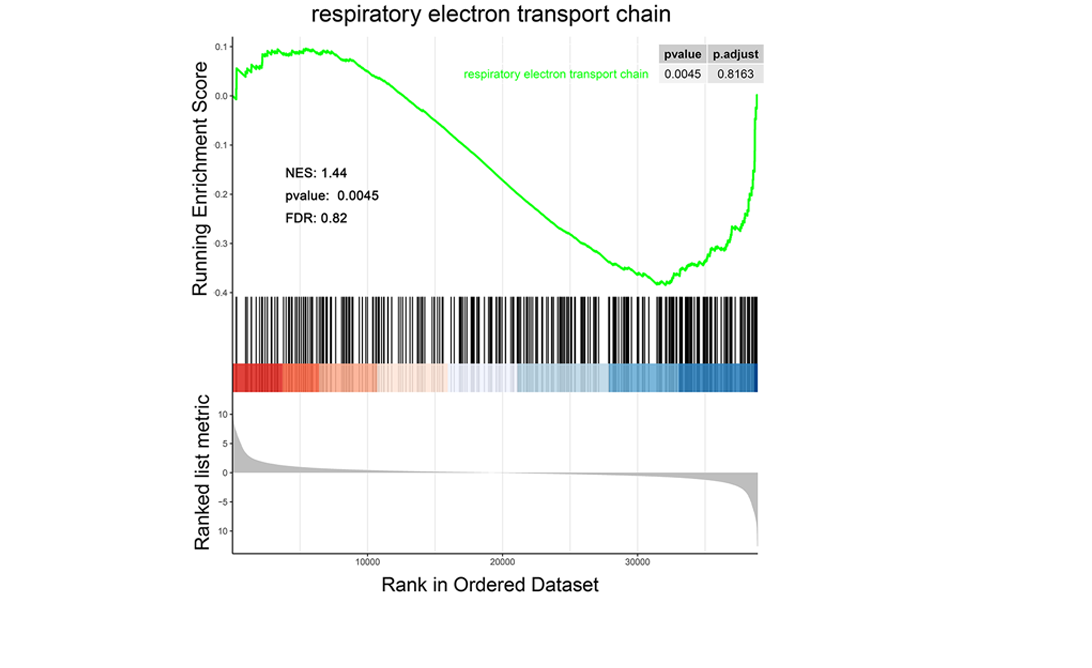


Figure S18. Respiratory electron transport chain based on GSEA.


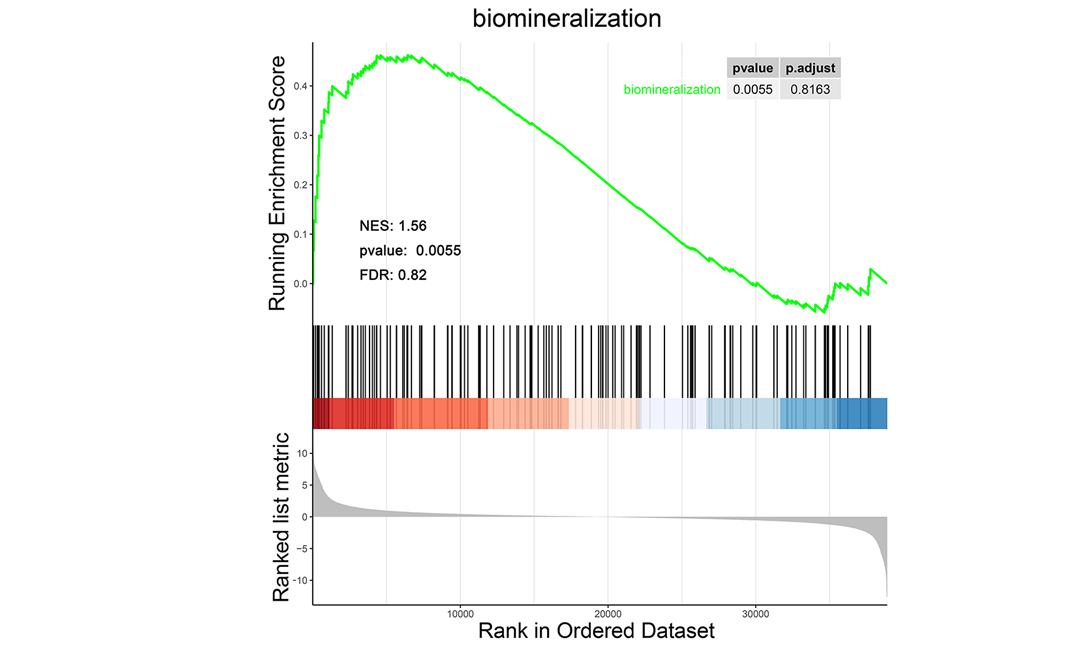


Figure S19. Biomineralization based on GSEA.


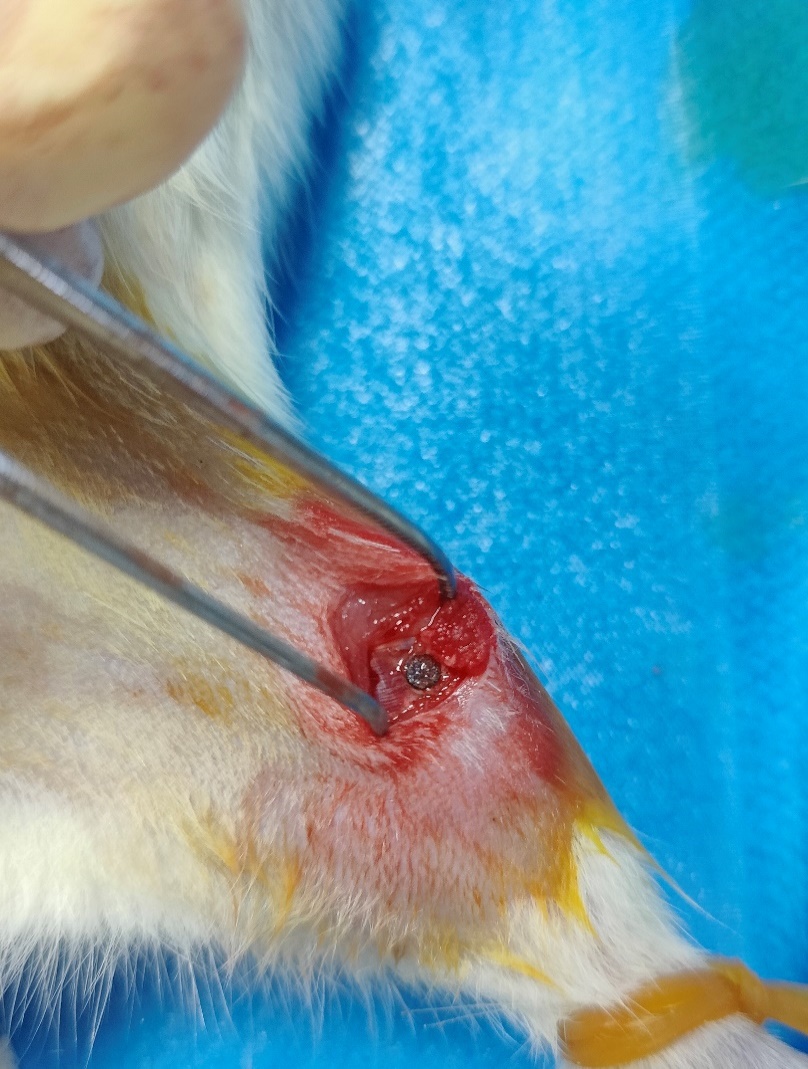


Figure S20. Physical image from the animal experiment.


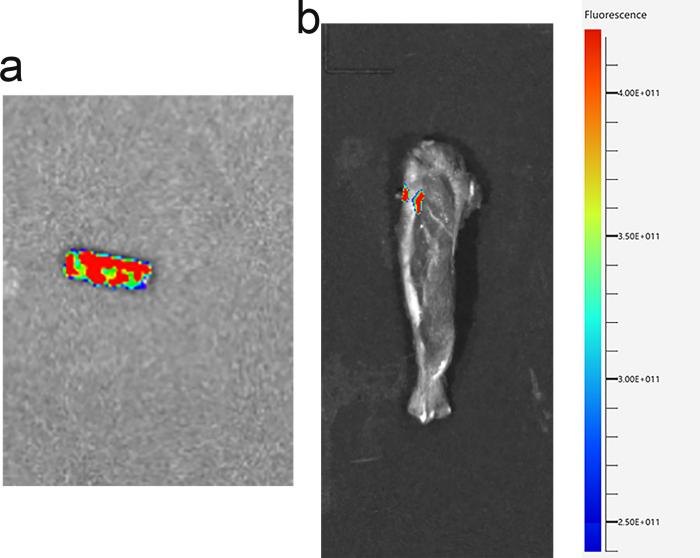


Figure S21. In situ in vivo imaging of US excited DTO/BTO to produce singlet oxygen (excitation and emission peaks of 504 nm and 525 nm, respectively).


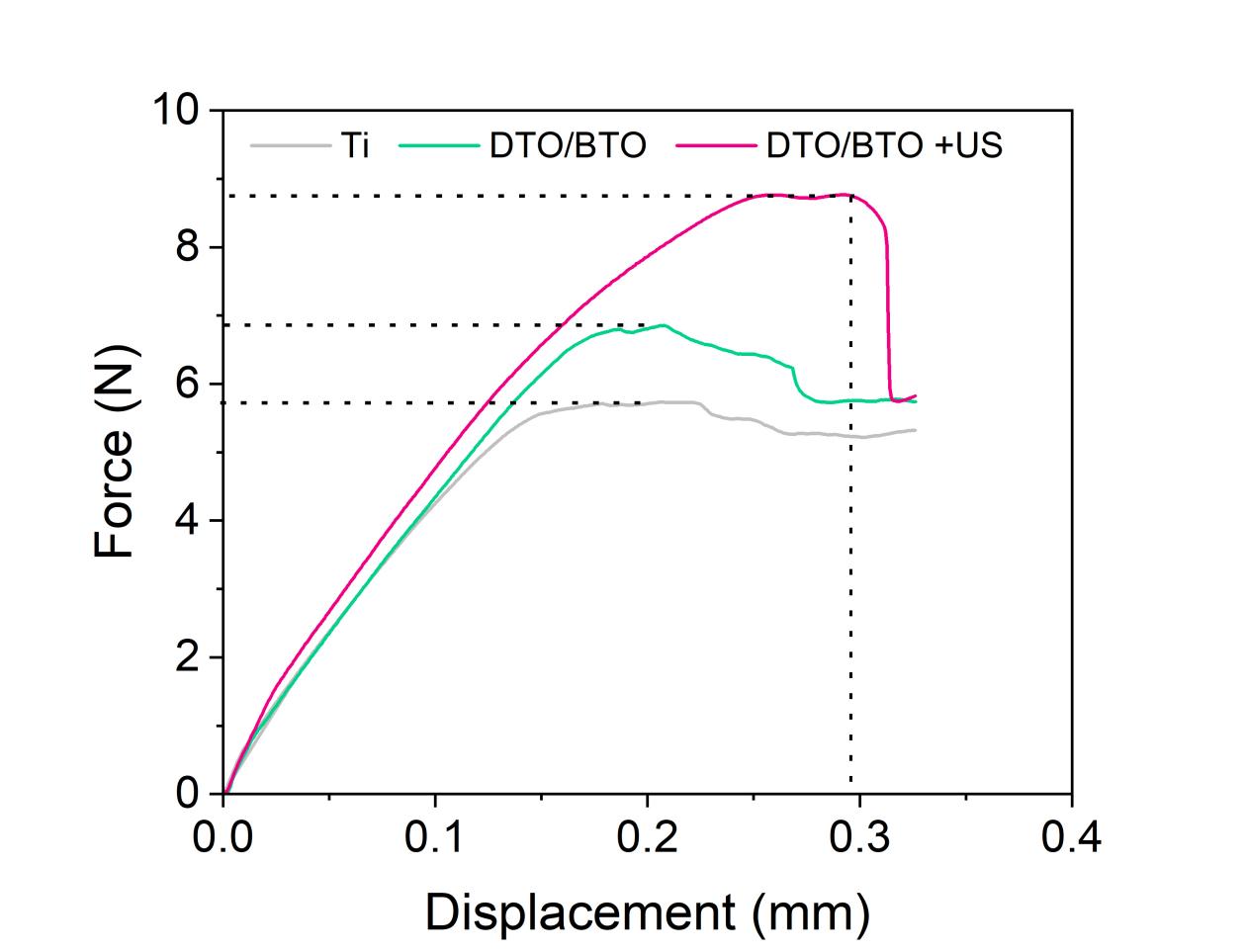


Figure S22 . The pull-out forces of the different samples.


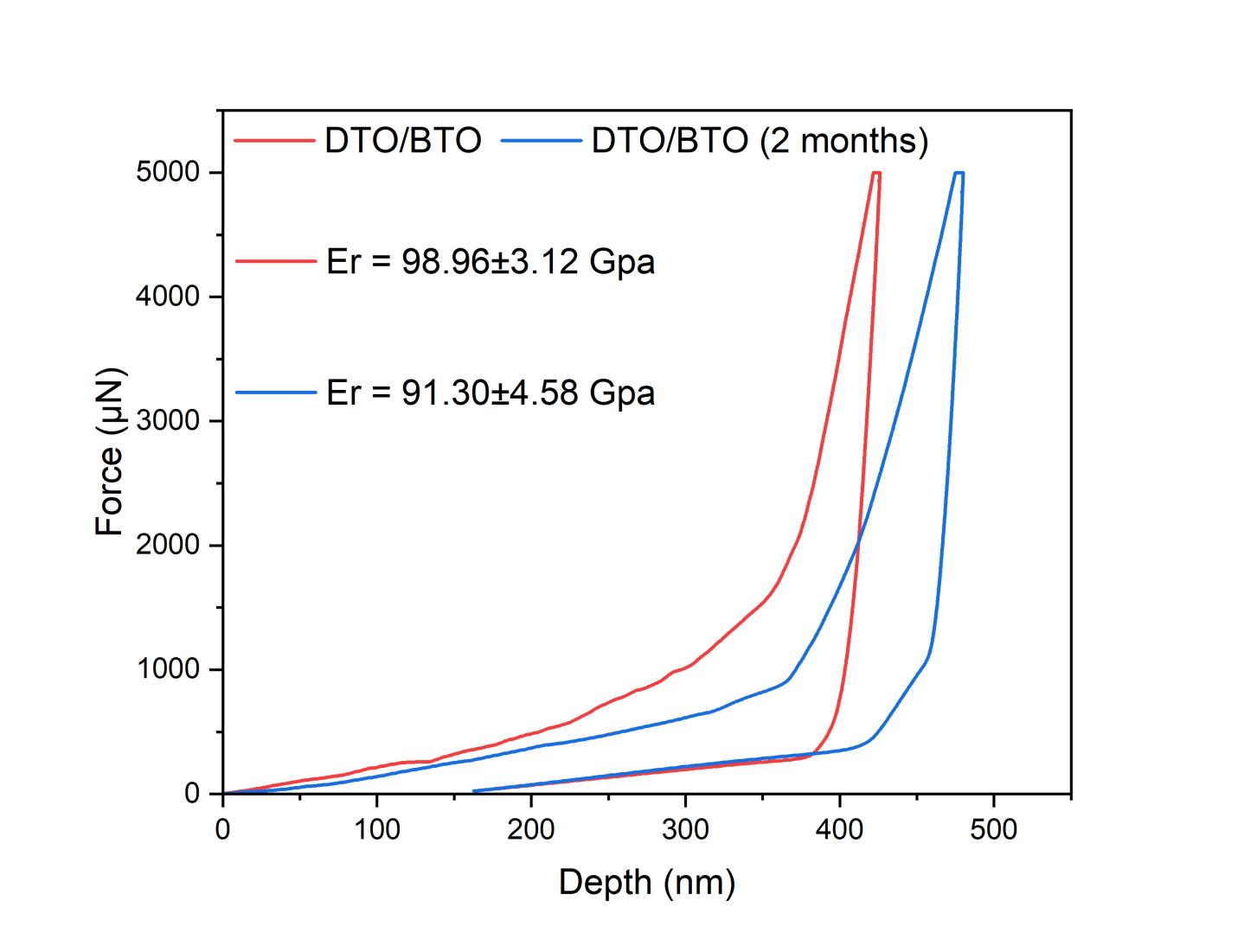


Figure S23. Elastic modulus of DTO/BTO and DTO/BTO (2 months); the results exhibited the long-term stability and good mechanical property of DTO/BTO.


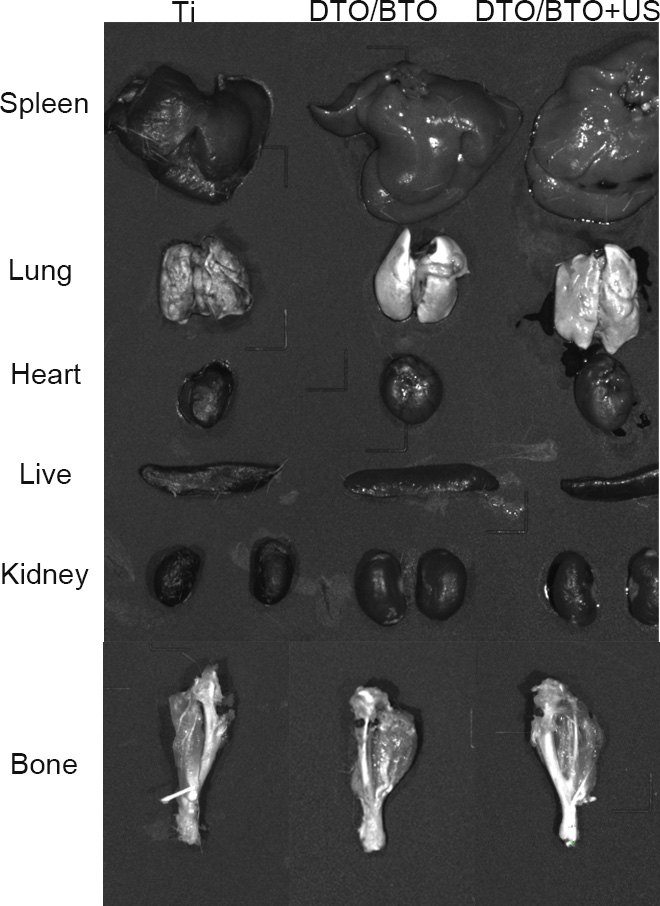


Figure S24 .In vivo imaging of major organs (bone marrow, heart, liver, spleen, lung, kidney) in different groups of SD rats after 28 days of treatment.


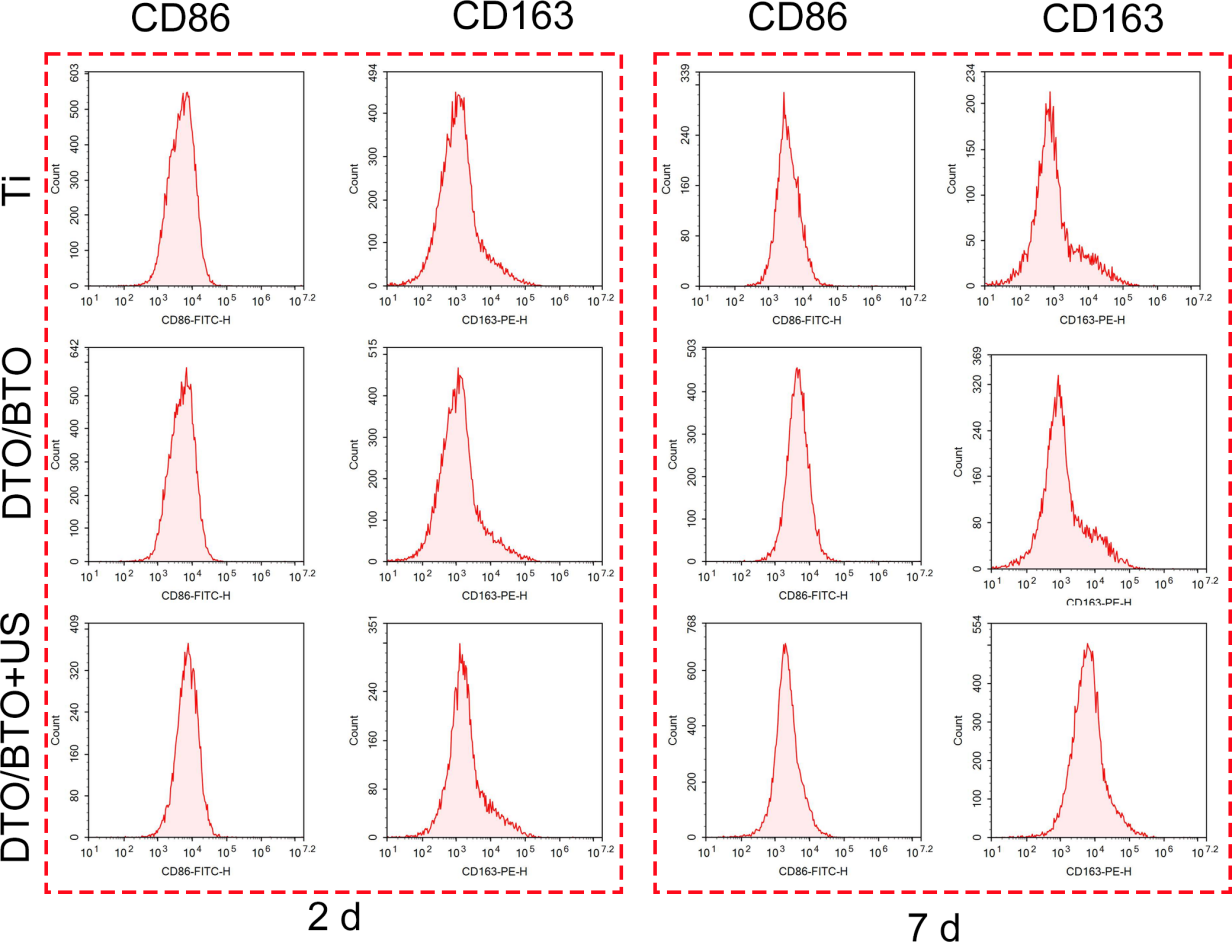


Figure S25.After DTO/BTO with US treatment in bone defects, flow cytometry was applied to detect CD86 labeled M1-type macrophages,CD163 labeled M2-type macrophages.

To investigate the local immune response, we performed flow cytometry to analyze macrophage polarization around the implant site. The results showed that on Day 2, CD86—a representative marker of pro-inflammatory M1 macrophages—was expressed at significantly higher levels than CD163, a marker of anti-inflammatory M2 macrophages. However, by Day 7, this pattern reversed, with an increased presence of M2 macrophages.

These results suggest that in the early stage of infection, due to the presence of bacteria and necrotic tissue, the local immune environment is dominated by M1 macrophages, which play a key role in bacterial clearance. Following ultrasound treatment, a phenotypic shift from M1 to M2 macrophages occurred, indicating a transition from a pro-inflammatory to a reparative, anti-inflammatory state that contributes to tissue regeneration and bone healing.


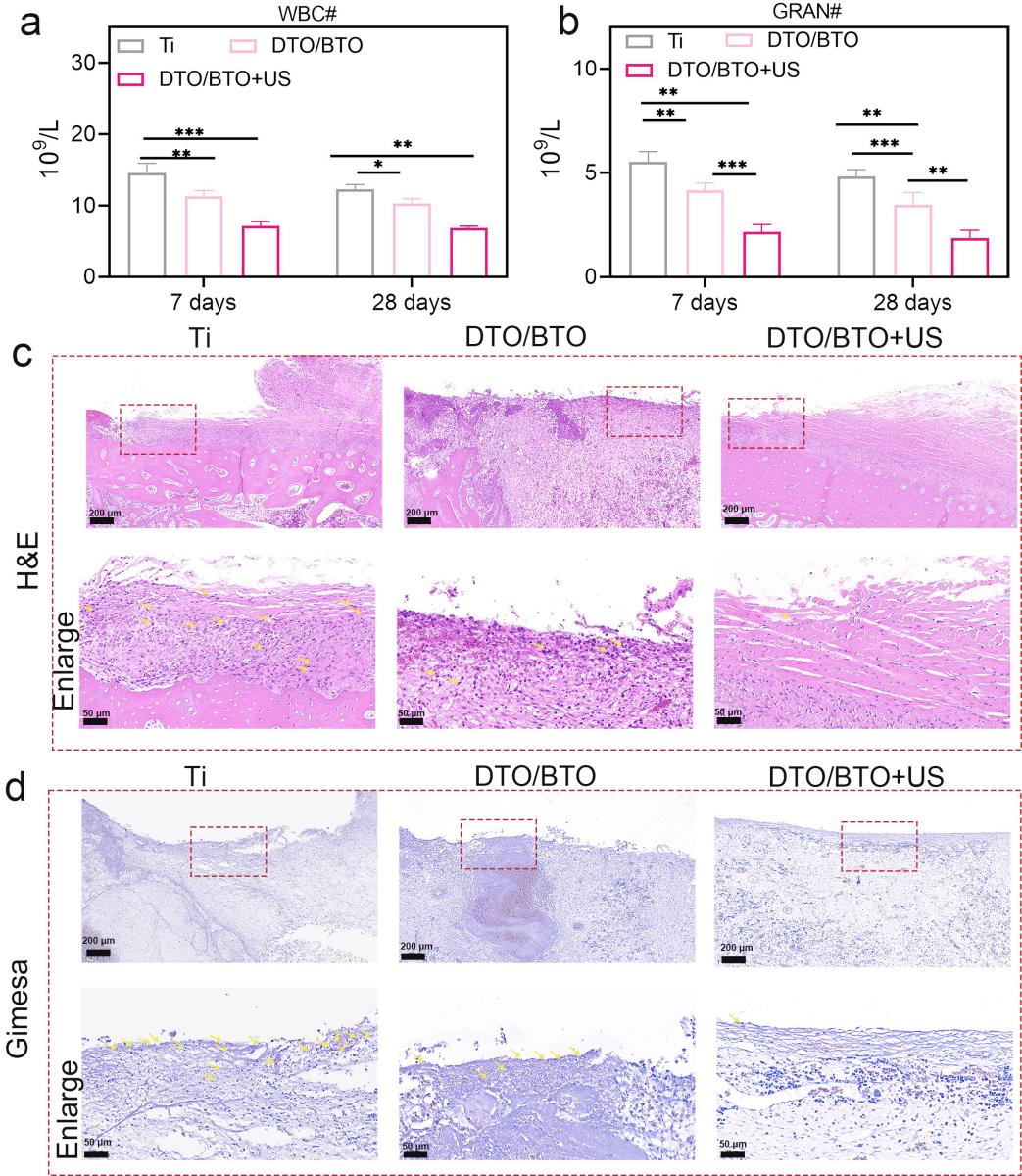


Figure S26. In vivo antibacterial experiment of rabbit. a) WBC and (b) gran counts from blood from the different groups tested at 7 days and 28 days post-surgery.(The error bars indicate means ± SD, n = 3. *p < 0.05, **p < 0.01, ***p< 0.001). (c) H&E staining of different groups on 7days and (d) Giemsa staining of different groups on 7days.( n=3, (*) p <0.05, (**) p <0.01, (***) p <0.001 ).


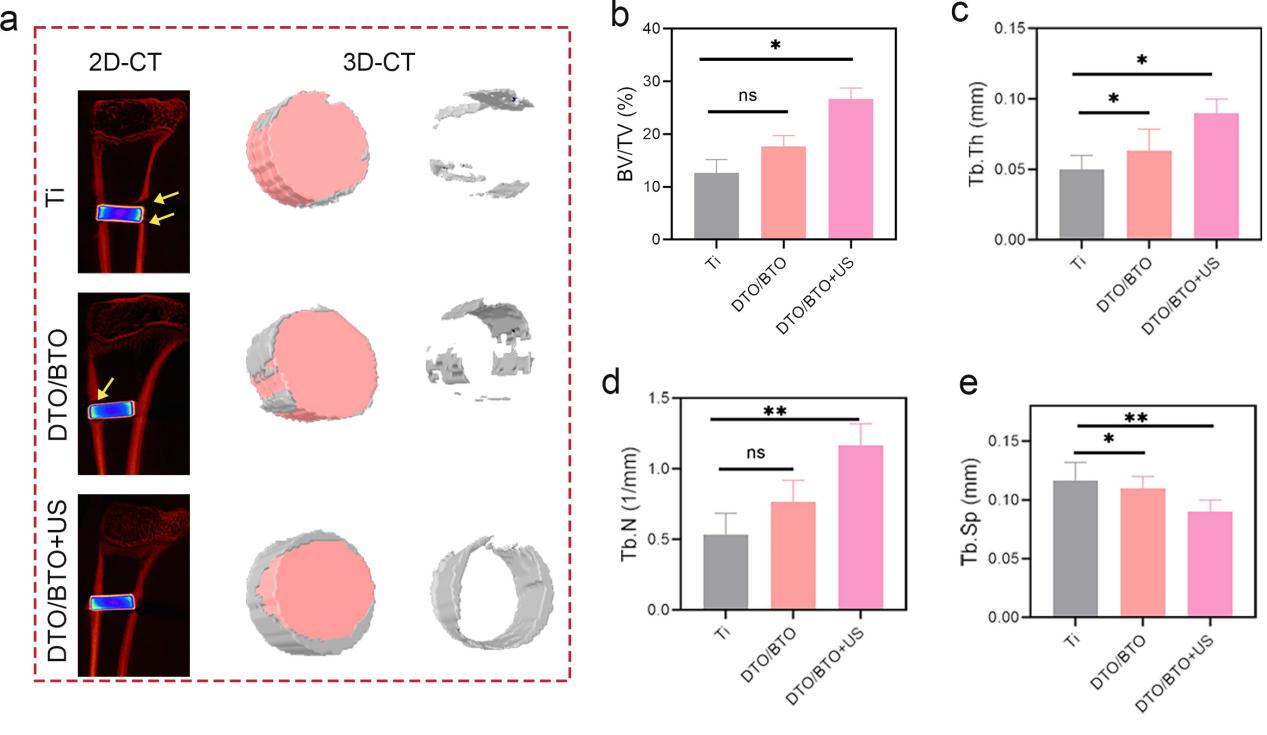


Figure S27. Bone formation evaluation in vivo of rabbit. (a) 2D and 3D micro-CT reconstructed images of the Ti, DTO/BTO and BTO/DTO +US. Quantitative analysis of (b) new bone volume fraction (BV/TV), (c) trabecular thickness, (d) trabecular number (Tb. N) and (e) trabecular separation (Tb. Sp). ( n=3, (*) p <0.05, (**) p <0.01, (***) p <0.001 ).

Table 1. The primer sequences of the MSCs in the qRT-PCR gene expression

analysis.

| gene | Primer sequence (5’ to 3’) |
| --- | --- |
| GAPDH | F: GACACGGACAGGATTGACAGAT |
|  | R: TGCCAGAGTCTCGTTCGTTATC |
| RUNX2 | F: TGATGGTGTTGACGCTGATGG |
|  | R: TCGCCAGACAGACTCATCCA |
| OCN | AGAACAGACAAGTCCCACACAG |
|  | GCCAGCAGAGTGAGCAGAGA |
| Col1 | GTGTGCGATGGCGTGCTAT |
|  | TGACTTCTGCGTCTGGTGATAC |
